# Supplementary material for: Endogenous tyrosinase-catalyzed therapeutics
Source: Nat Commun. 2025 Jul 12;16:6463. doi: 10.1038/s41467-025-61799-7 (PMC12255739; doi:10.1038/s41467-025-61799-7)
Supplement: Supplementary file 1 — Supplementary Information [file 41467_2025_61799_MOESM1_ESM.pdf]

Supplementary Information

Endogenous Tyrosinase-Catalyzed Therapeutics

Yawen You[1, 2, 3], Zhaochen Guo[4], Yixin Wang [1, 2, 3], Sichen Yuan[1, 2, 3], Quanyin Hu[1, 2, 3, *]

[1] Pharmaceutical Sciences Division, School of Pharmacy, University of Wisconsin-Madison, Madison, WI 53705, USA.
[2] Carbone Cancer Center, School of Medicine and Public Health, University of Wisconsin-Madison, Madison, WI 53705, USA.
[3] Wisconsin Center for NanoBioSystems, School of Pharmacy, University of Wisconsin-Madison, Madison, WI 53705, USA.
[4] Department of Biochemistry, College of Agriculture and Life Sciences, University of Wisconsin-Madison, Madison, WI 53706, USA.

* Corresponding author: Quanyin Hu. E-mail: qhu66@wisc.edu

Reagents and antibodies

(S, R, S)-AHPC-PEG$_2$-N$_3$, (S, R, S)-AHPC-PEG$_3$-N$_3$, and (S, R, S)-AHPC-PEG$_4$-N$_3$ were purchased from MedChemExpress (Princeton, NJ, USA). Epoxomicin was bought from MedChemExpress (Monmouth Junction, NJ, USA). 1,3-Bis (2,4-dihydroxy phenyl) propane-1-one, (2S, 4R)-1-((S)-2-amino-3,3-dimethyl butanol)-4-hydroxy-*N*-(4-(4-methyl thiazole-5-yl) benzyl) pyrolidine-2-carboxamide hydrochloride (VHL ligand), Boc-Tle-OH, kojic acid, cis-*N*-(tert-Butoxycarbonyl)-4-hydroxy-L-proline, (4-(4-Methylthiazol-5-yl)phenyl) methanamine, (2S,4S)-1-(tert-butoxycarbonyl)-4-hydroxypyrrolidine-2-carboxylic acid were purchased from AmBeed (Arlington Hts, IL, U.S). Iodocopper (CuI) was purchased from Combi Blocks (San Diego, CA, U.S.). Phloroglucinol, ethynylbenzene, and L-tyrosine were bought from the Tokyo Chemical Industry (TCI). Tyrosinase (T3824), α-Melanocyte stimulating hormone, and 14-azido-3,6,9,12-tetraoxatetradecanoic acid solution were bought from Sigma-Aldrich (St. Louis, MO, U.S). Bis(triphenylphosphine) palladium (II) dichloride (Pd (PPh3)2Cl2), sodium azide (NaN$_3$), 4-iodophenol, O-(7-Azabenzotriazol-1-yl)-*N*, *N*, Nzzhlxy, Nzzhlxy-tetramethyluronium hexafluorophosphate (HATU), and *N*-tertbutyloxycarbonyl-6-amino-hexanoic acid were bought from Chem-Impex (Wood Dale, IL, U.S). Propargyl bromide, copper sulfate anhydrous (CuSO$_4$), sodium ascorbate, *N*, *N*-Diisopropylethyl amine, trifluoroacetic acid, triethanolamine, ethynyl trimethyl silane, potassium carbonate (K$_2$CO$_3$), and Pierce™ Protein A/G Magnetic Beads (88802) were bought from Thermo Fisher Scientific (Waltham, MA, U.S). Dead Cell Apoptosis Kit with Annexin V FITC and PI (640914) was acquired from BioLegend (San Diego, CA, U.S.). Cell Counting Kit-8 (CCK-8) kit was acquired from APExBIO (Houston, TX, U.S.). Creatinine (urinary) Colorimetric Assay Kit (500701), Aspartate Aminotransferase Colorimetric Activity Assay Kit (701640), and Alanine Transaminase Colorimetric Activity Assay Kit (700260) were purchased

from Cayman (Ann Arbor, MI, U.S). CoraLite®594 TUNEL Assay Apoptosis Detection Kit (PF00009) was bought from Proteintech (Rosemont, IL, U.S.). VitroView™ Fontana-Masson Stain Kit (VB-3020) was purchased from Vitro Vivo Biotech (Rockville, MD, U.S.). The Amplite™ Colorimetric Tyrosinase Assay Kit (3321821) was purchased from AAT Bioquest. The mouse Von Hippel Lindau/VHL Antibody (VHL40) (sc-135657) was purchased from Santa Cruz Biotechnology (Dallas, TX, U.S.). Tyrosinase Recombinant Rabbit Monoclonal Antibody (JA52-11), MITF (D5G7V) Rabbit mAb (12590), HRP-anti-β-actin (ab49900) antibodies and secondary antibodies used in the western blotting assay were obtained from Abcam (Cambridge, UK). All antibody dilutions were performed according to the manufacturer's guidance.

Measurements and characterizations

^{1}H NMR spectrum and ^{13}C NMR spectrum were recorded via a Bruker Avance III HD 400 MHz NMR instrument (AV-400) NMR instrument. Liquid Chromatography Mass Spectra (LC-MS) measurements were performed on a Bruker MaXis time-of-flight spectrometer. Fluorescence spectra were detected by a QuantaMaster Model C-60/2000 Spectrofluorimeter. A Nikon Intensilight fluorescence microscope was used for bright-field microscopy characterization. Agilent Infinity II was used to perform the high-performance liquid chromatography (HPLC) analysis. A Nikon AXR Confocal Microscope was used to acquire the confocal laser scanning microscopy (CLSM) characterization. The ThermoFisher Attune Cell Analyzer collected the flow cytometry data.

Quantitative analysis of TYR in different cell lines

Normal human melanocyte, A375, and B16F10 cells were seeded at a density of 2×10^5 cells/well into 6-well plates for 24 h. Cells were washed thrice with PBS. After trypsinization and resuspension in the tyrosinase substrate-containing working solution, cells were subjected to quantification by the tyrosinase assay kit protocol. Absorbance at 510 nm was determined with a microplate reader.

Wash-out experiment

To study the long-lasting degradation efficacy against TYR, the degradation efficacy of TYR-catalyzed TYR degraders in both A375 and B16F10 cells was tested using the western blot assay. A375 and B16F10 cells were seeded at a density of 2×10^5 cells/well in DMEM with 10% FBS. After the 24 h incubation, cells were then exposed to VH032-Azi3 (0.1 μM) + Alk-TIn (0.1 μM) + SA (0.5 μM) or DMSO-containing DMEM for various drug-incubation times and continuous monitoring time points. After 12 h of incubation, cells were washed with ice-cold PBS, harvested in RIPA lysis buffer, and lysed on ice for 30 minutes. Cells were then centrifuged for 30 minutes at 16,200 g. The protein contents in the supernatants were quantified using a bicinchoninic acid (BCA) assay. Following protein extraction, supernatants were prepared with a loading buffer and heated at 95 °C for 15 minutes. Each sample (15 μg of protein) was loaded into a 12% SDS-polyacrylamide gel for protein separation. Afterward, proteins were transferred to PVDF membranes, blocked with 5% nonfat milk in PBST, and incubated with primary antibodies against TYR (1:1000) at 4 °C overnight. Followed by a 1 h room temperature incubation with goat anti-rabbit IgG H&L (HRP) secondary antibodies (1:10,000). Protein bands were visualized using electrochemiluminescence (ECL) and quantified with ImageJ software.

Linker conformations

RDKit that generates 10,000 conformations with a threshold value larger than 1.0, and 1584 conformers was selected according to the energy ranging from low to high (**Supplementary Fig. 21a**).

Preparation of the starting structure

Two complex structures (TYR (AlphaFoldDB:P11344) and VHL (PDB code: 8bdi) were initially downloaded from the RCSB PDB to model the ternary structure for mTYR, VHL E3 ligases, and PROTAC. Given that no crystal structure is available for the mTYR, Therefore, we employed Autodock vina[1] to carry out molecule docking to construct a ligand-mTYR complex (**Supplementary Fig. 21b**). Afterward, PatchDock[2] was used for protein-protein global docking to construct a complex model (**Supplementary Fig. 22**), with a distance constraint between the binding pockets of the two ligands. The distance constraint range was determined according to the distribution of distances between two generated linker conformations (**Supplementary Fig. 21a**). Afterward, we used ROSETTA2020 to parameterize the ligand in VHL and mTYR for later local docking by the command as follows:

$ROSETTA3/scripts/python/public/molfile_to_params.py -n NM1 -p NM1 NM1.mol2
$ROSETTA3/scripts/python/public/molfile_to_params.py -n NM2 -p NM2 NM2.mol2
$ROSETTA3/bin/docking_prepack_protocol.linuxgccrelease -s Complex.pdb -use_input_sc -extra_res_fa NM1.params NM2.params

Rosetta protein−protein docking

10,000 diverse binding modes were output by the docking_protocol.mpi_linux_gcc release program. The flag "-partners AX_CY" specifies that the small-molecule ligands must move together with their paired proteins. The "-dock_pert" flag was employed to define the search space. Afterward, the InterfaceAnalyzer module in ROSETTA was used to evaluate the decoy with a distance between two linker atoms smaller than 21 Å. Next, this result was filtered with the dG_separated/dSASA*100 ≤ 1.0 and packsat > 0.5. Finally, 289 decoys were selected to build the ternary models.

Ternary models

The RMSD of the stubs in the given linker conformer relative to their location was evaluated in a docked model by a Python script[3]. The script will build and output the complete model of the ternary complex while the RMSD of the stubs passes a specified cutoff value. Finally, we selected the best result score of the docking result and the energy of the ligand conformer for molecular dynamics simulation (**Supplementary Fig. 23a**).

Molecular dynamics simulation

The stable ternary structure was filtered by molecular dynamics (MD) simulation. The TIP3P water model was employed to build the water box before the MD simulation; concomitantly, chlorine or

sodium ions were added to neutralize the system. AMBER package[4] was employed to run the MD simulations with leaprc.protein.ff14SB[5] as the force field for the protein. The electrostatic potential was calculated at the B3LYP/6-311G* level by Gaussian 09[6] for PROTACs. Then the restrained electrostatic potential[7] was computed by Antechamber[8].

We first performed energy minimization to obtain a low-energy starting conformation for the subsequent MD simulations. 4000 steps of the steepest descent method were employed, followed by 6000 steps of the conjugate gradient method. The whole system (protein, ligand, water, ions) was minimized, and the solutes (protein and ligand) were further minimized. With the Langevin thermostat applied, the system was heated under canonical ensemble from 0 to 310 K for 300 ps, with the force constant for the harmonic restraint set to be 10.0 kcal mol Å. The system was then equilibrated for 10 ns under NPT conditions (constant pressure = 1.0 bar). The relaxation time for the barostat bath was 2.0 ps. Finally, the production simulation was run for 100 ns under NPT with periodic boundary conditions. SHAKE algorithm constrained the bonds connection of hydrogen atoms for 2 fs time step. Particle-mesh Ewald (PME) method[9] was used to deal with the long-range electrostatics. The cut-off value for short-range interactions was set to be 10.0 Å.

The pairwise RMSD (root mean square deviation) (**Supplementary Fig. 23b**) of the atoms was calculated for analyzing the stability of the complexes during the simulation and ensuring the basic analysis of trajectories of the sampling method by MDAnalysis[10] python package. Then, the pairwise RMSD matrix was used to cluster the trajectory through the Density Peak method[11]. The trajectory was clustered into two groups. The representative structure in the main group was selected as the most stable mode for detailed analysis.

Western blot analysis for UPS-dependent mechanism investigation

As for the Epox-involved treatments, A375 and B16F10 cells were first treated with 1 mL of medium containing Epox (0.2 μM) for 1 h after a 24 h incubation, and then the medium was replaced with 1 mL of medium containing the corresponding small molecules (0.1 μM) and Epox (0.2 μM). After incubation for 12 h, cells were washed with PBS and lysed with RIPA lysis buffer containing phenylmethylsulfonyl fluoride (PMSF, 1 mM) and the phosphatase inhibitor cocktail for 30 min. The lysates were centrifuged at 13,800 g for 10 min at 4 °C, followed by quantification of the total proteins in the supernatant through the bicinchoninic acid assay (BCA) assay. Afterward, the proteins in the supernatant were mixed with the loading buffer and heated at 95 °C for 15 min. Equal amounts of proteins from different samples were loaded into a 12% SDS-polyacrylamide gel (15 μg protein per lane) to separate target proteins. After separation, proteins were transferred to polyvinylidene fluoride (PVDF) membranes before the membranes were blocked with 5% nonfat milk in PBST. Then proteins were incubated with primary antibodies overnight at 4 °C, followed by incubation with the goat-anti-rabbit IgG H&L (HRP) secondary antibodies at room temperature for 1 h. Protein bands were visualized using electrochemiluminescence (ECL) via an Azure 280 chemiluminescent imaging system and quantified with ImageJ software.

The long-term monitoring of skin hyperpigmentation

Male nude mice (5–6 weeks) were used to establish a skin hyperpigmentation model. An ethanol solution of 15% DHA (w/w) and 3% erythrulose (w/w) was applied to the backs of mice in a square pattern, followed by a UV irradiation (300 μW/cm² at 10 W, Farmingdale, NY, USA) for 15 minutes. Three days later, these mice were randomly assigned into two groups (n=6 per group) for subsequent treatments, including a blank control and VH032-Azi3 + Alk-TIn + SA creams at a concentration of 0.03% (w/w).To explore the long-term treatment efficacy of in-situ formed DeTYR-3, we extended the evaluation timeline on the skin hyperpigmentation mouse model after various treatments for 30 days.

Digital image analysis for histological quantification of melanin

For the identification of melanin-equivalent pixels in the skin tissues, we used Fontana-Masson stained and analyzed by ImageJ.[12] Each treatment group was randomly assigned three Fontana-Masson staining slides to obtain Normalized hyperpigmentation levels by Image J.

Skin penetration tests

To study the small molecule penetration performance of VH032-Azi3 and Alk-TIn cream, we modified them with the fluorescence groups FAM for VH032-Azi3 and Cy5.5 for Alk-TIn, respectively. A small molecule-based cream at a concentration of 1 mM was applied to the skin on the backs of mice for 24 hours. The control group was first dissolved in a small amount of DMSO, then diluted in PBS to a final concentration of 1 mM. After treatments, mice were euthanized, and skin samples were harvested, fixed in 10% neutral buffered formalin, embedded in paraffin, sectioned to 5 μm, and stained using H&E.

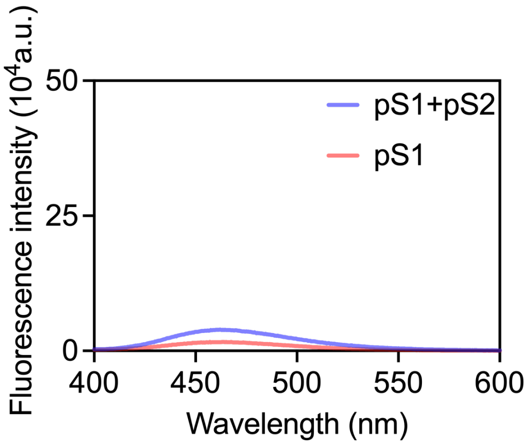

Supplementary Fig. 1. Fluorescence spectra of pS1 and pS1 + pS2. (a. u., arbitrary units).

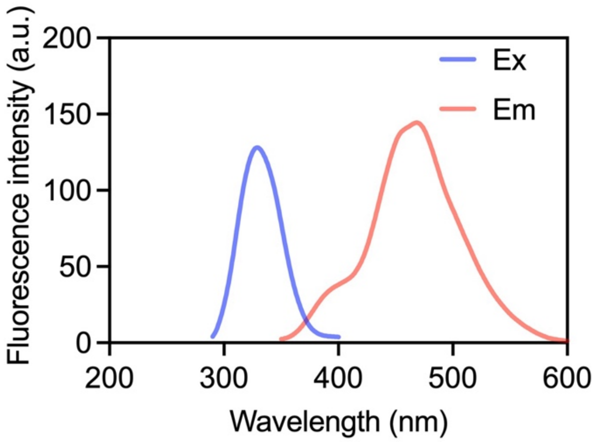

Supplementary Fig. 2. Excitation and emission of fluorescence (Fluo) in H_2O. Cycloaddition of pS1 and pS2 generated Fluo. (λex =330 nm and λem =470 nm). (a. u., arbitrary units).

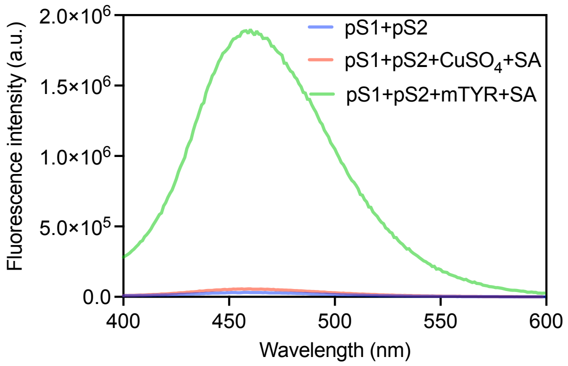

Supplementary Fig. 3. Fluorescence spectra of pS1 + pS2, pS1 + pS2 + $CuSO_4$ + SA and pS1 + pS2 + mTYR + SA. pS1, pS2 (10 μM), mTYR: mushroom tyrosinase (1mg/mL), $CuSO_4$ (8.35 μM) and SA (41.75 μM). (a. u., arbitrary units).

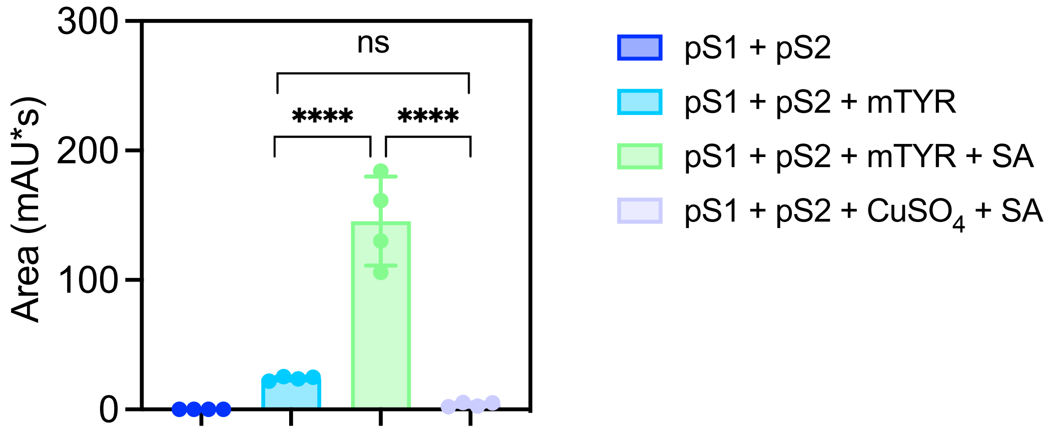

Supplementary Fig. 4. Quantitative analysis of high performance liquid chromatography analysis of Fluo that catalyzed by pS1 + pS2, pS1 + pS2 + mTYR, pS1 + pS2 + mTYR + SA, and pS1 + pS2 + $CuSO_4$ + SA. pS1, pS2 (10 μM), mTYR: mushroom tyrosinase (1mg/mL), $CuSO_4$ (8.35 μM) and SA (41.75 μM). Statistical analysis was performed using ONE-WAY variance (ANOVA) by Dunnett's multiple comparisons (****$P < 0.0001$). (n = 3 independent experiments).

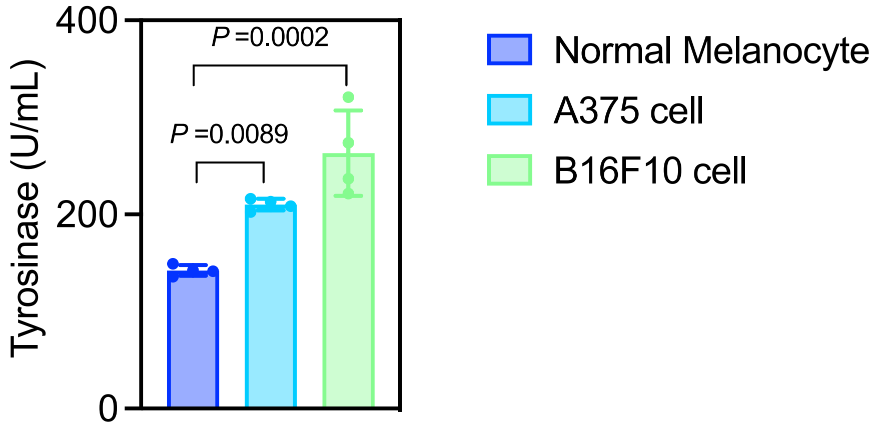

Supplementary Fig. 5. Quantitative analysis of TYR expression in normal melanocytes, A375, and B16F10 cells. Statistical analysis was performed using ONE-WAY variance (ANOVA) by Dunnett's multiple comparisons (**$P < 0.01$, ***$P < 0.001$). (n = 3 biological replicates).

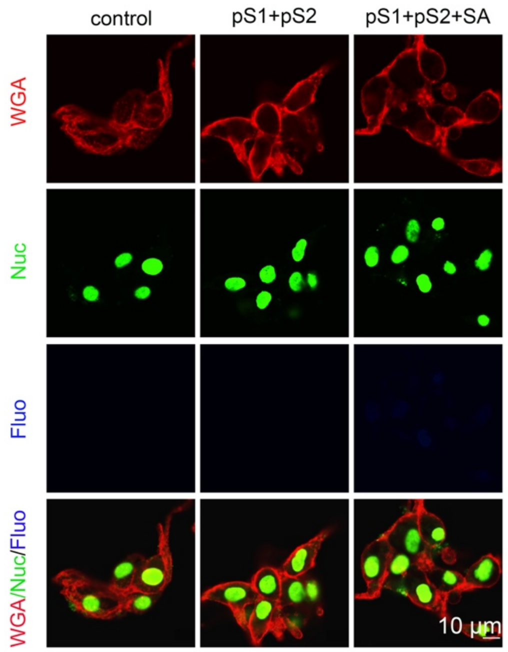

Supplementary Fig. 6. Representative confocal microscopy images of normal melanocytes treated with pS1 + pS2 and pS1 + pS2 + SA. (n = 3 independent experiments).

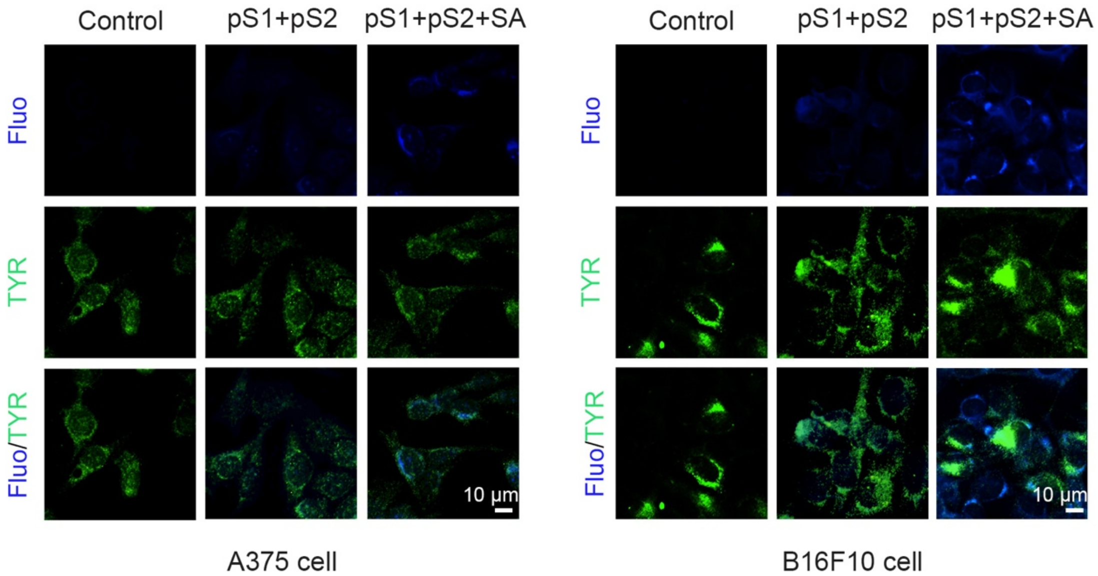

Supplementary Fig. 7. Representative confocal microscopy images of colocalization of “Fluo” fluorescence with TYR distribution in A375 and B16F10 cells treated with pS1 + pS2 and pS1 + pS2 + SA, respectively. (n = 3 independent experiments).

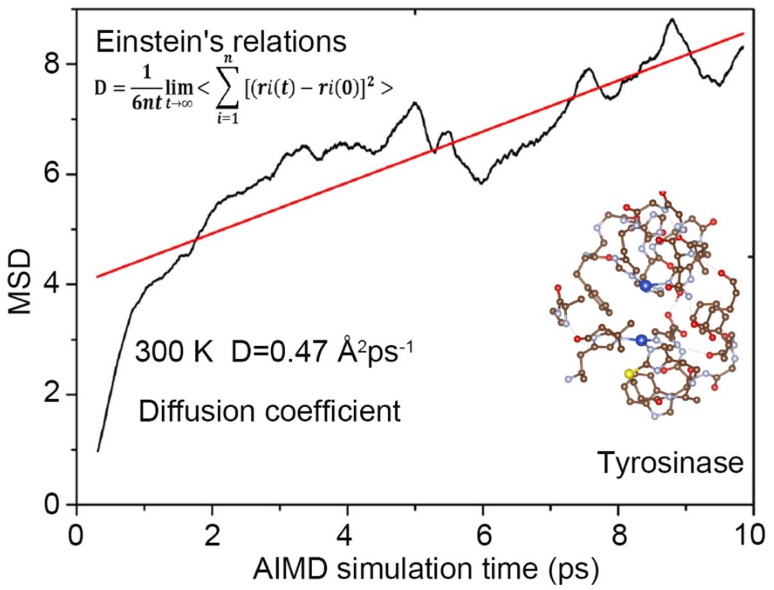

$$D = \frac{1}{6nt} \lim_{t \to \infty} < \sum_{i=1}^{n} [\boldsymbol{r_i}(t) - \boldsymbol{r_i}(0)]^2 >$$

Supplementary Fig. 8. The simulated diffusion coefficient (D) at 298 K during an equilibrium of the truncated model of the activation center of TYR by using AIMD simulation for 10 ps. MSD represents mean square deviation.

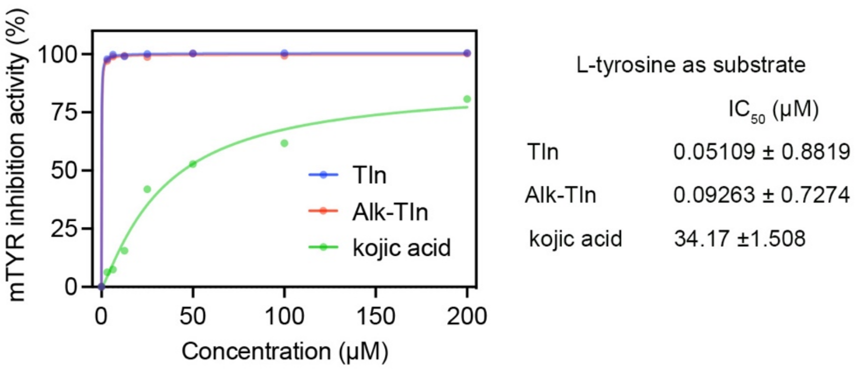

Supplementary Fig. 9. mTYR inhibition of TIn, Alk-TIn and kojic acid.

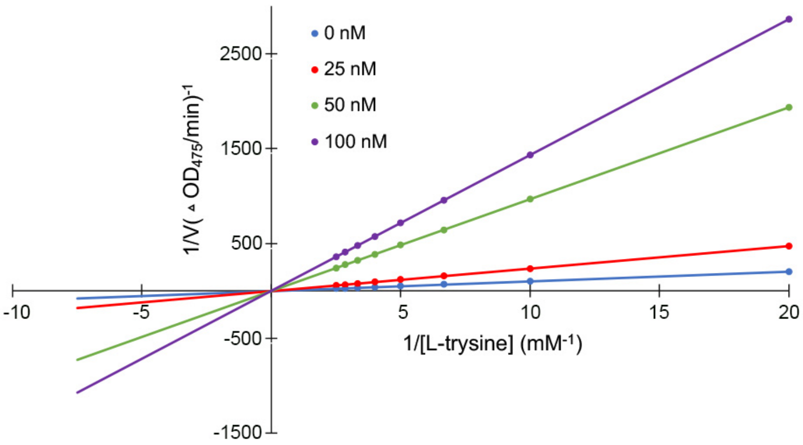

Supplementary Fig. 10. mTYR kinetic characteristic curves of Alk-TIn at different concentrations.

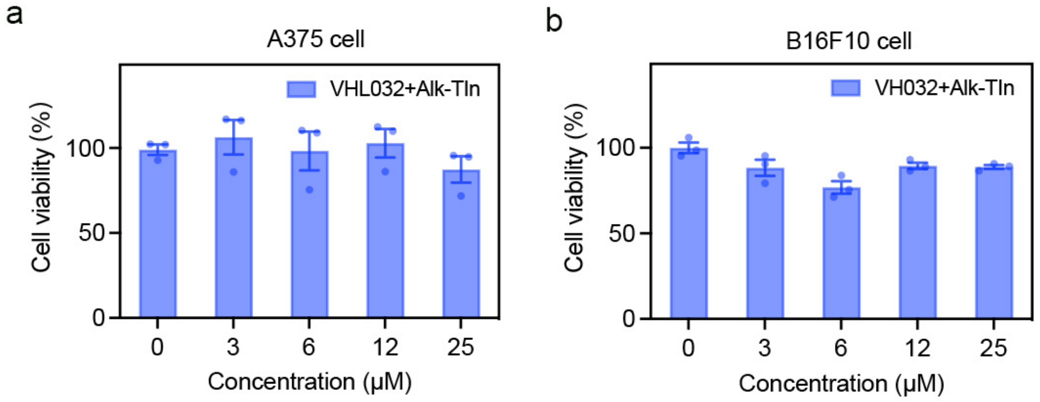

Supplementary Fig. 11. The viability of A375 (**a**) and B16F10 (**b**) cells treated with different concentrations of VH032 + Alk-TIn through a CCK-8 assay. Data are presented as Mean ± standard error (SEM), (n = 3 biological replicates).

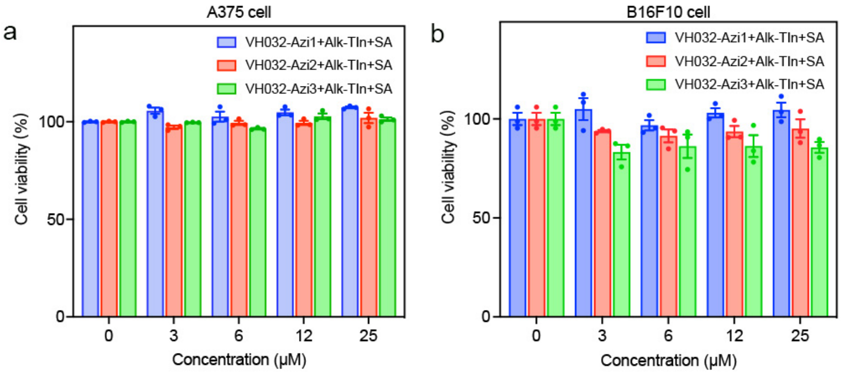

Supplementary Fig. 12. The viability of A375 (**a**) and B16F10 (**b**) cells treated with different concentrations of VH032-Azi1 + Alk-TIn + SA, VH032-Azi2 + Alk-TIn + SA and VH032-Azi3 + Alk-TIn + SA through a CCK-8 assay. Data are presented as Mean ± standard error (SEM), (n = 3 biological replicates).

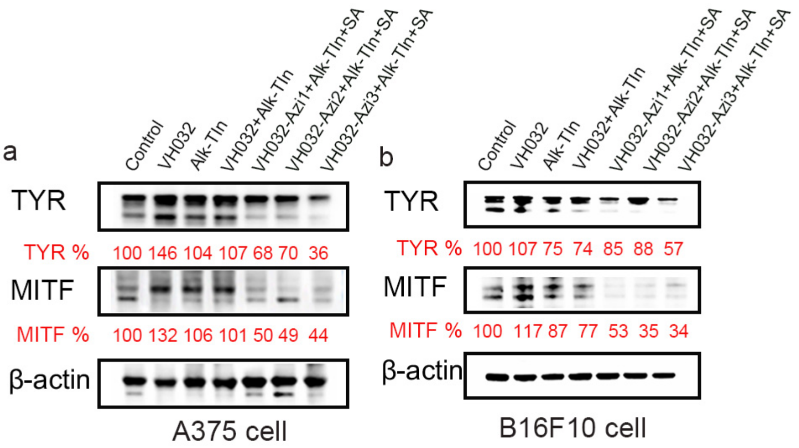

Supplementary Fig. 13. Western blot analysis of TYR and MITF in A375 (**a**) and B16F10 (**b**) cells treated with VH032, Alk-TIn, VH032 + Alk-TIn, VH032-Azi1 + Alk-TIn + SA (0.5 μM), VH032-Azi2 + Alk-TIn + SA (0.5 μM) and VH032-Azi3 + Alk-TIn + SA (0.5 μM) for 12 h. The concentrations of VH032, VH032-Azi, and Alk-Tin are 0.1 μM.

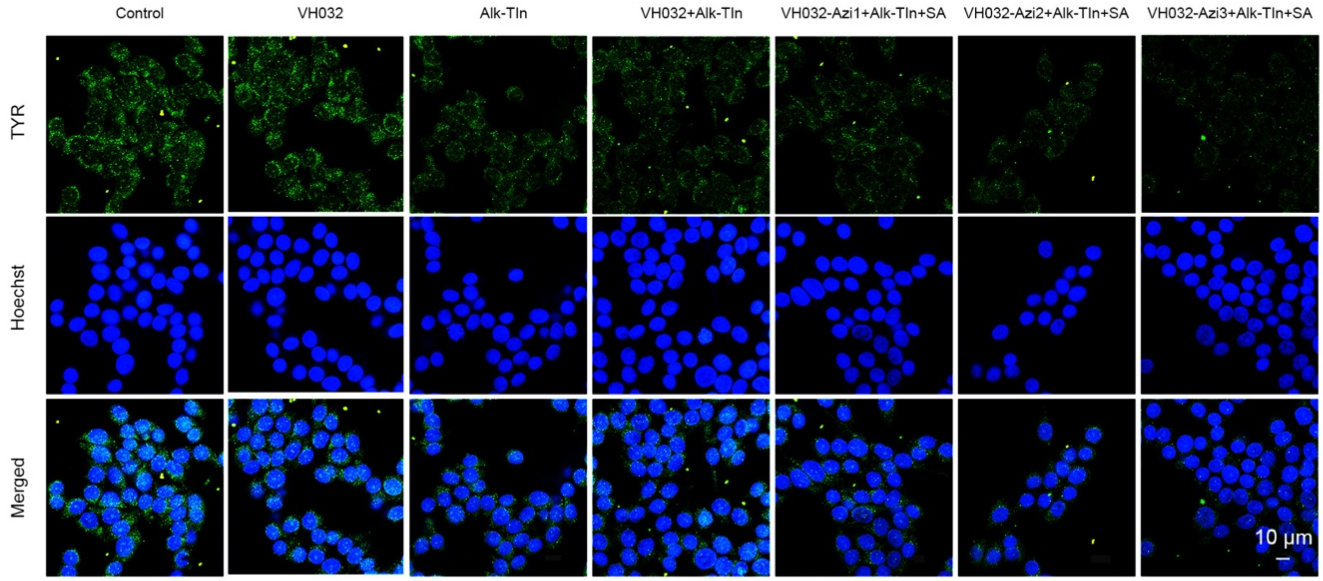

Supplementary Fig. 14. Representative confocal images of immunocytochemical staining of TYR in A375 cells. (n = 3 independent experiments).

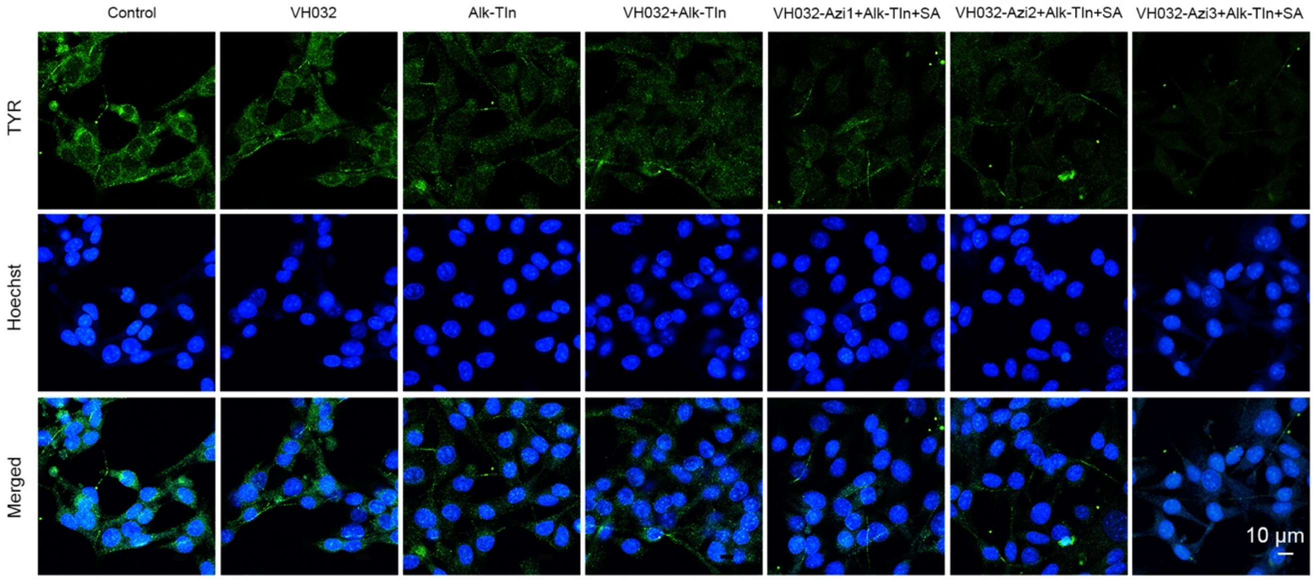

Supplementary Fig. 15. Representative confocal images of immunocytochemical staining of TYR in B16F10 cells. (n = 3 independent experiments).

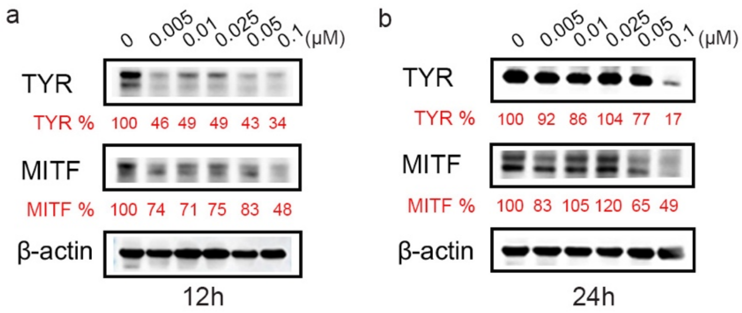

Supplementary Fig. 16. Western blot analysis of TYR and MITF in A375 cells treated with VH032-Azi3 + Alk-TIn + SA under different concentrations for 12 h (**a**) and 24 h (**b**).

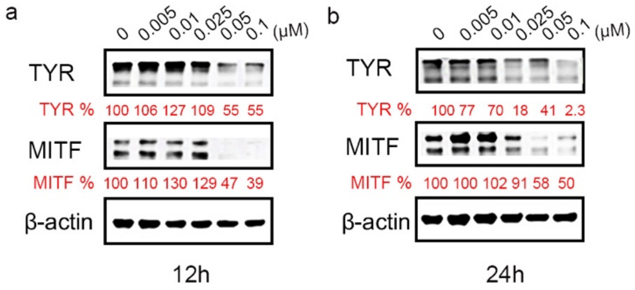

Supplementary Fig. 17. Western blot analysis of TYR and MITF in B16F10 cells treated with VH032-Azi3 + Alk-TIn + SA under different concentrations for 12 h (**a**) and 24 h (**b**).

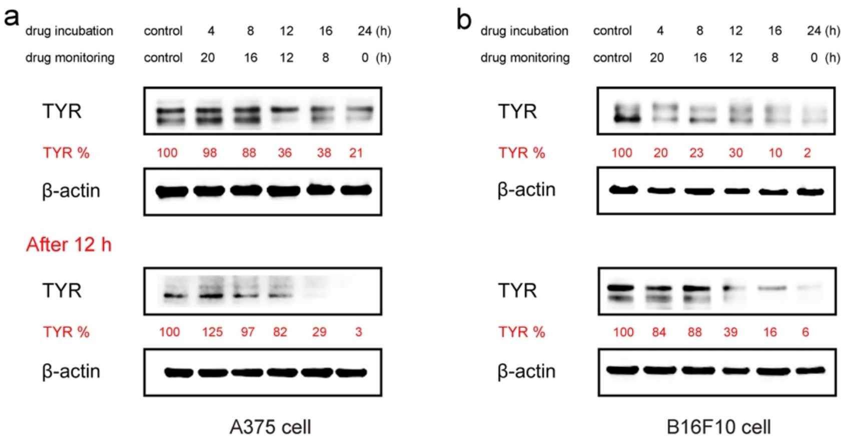

Supplementary Fig. 18. Western blot analysis of continuously degraded TYR in A375 (**a**) and B16F10 (**b**) cells treated with VH032-Azi3 (0.1 μM) + Alk-TIn (0.1 μM) + SA (0.5 μM) at different time points.

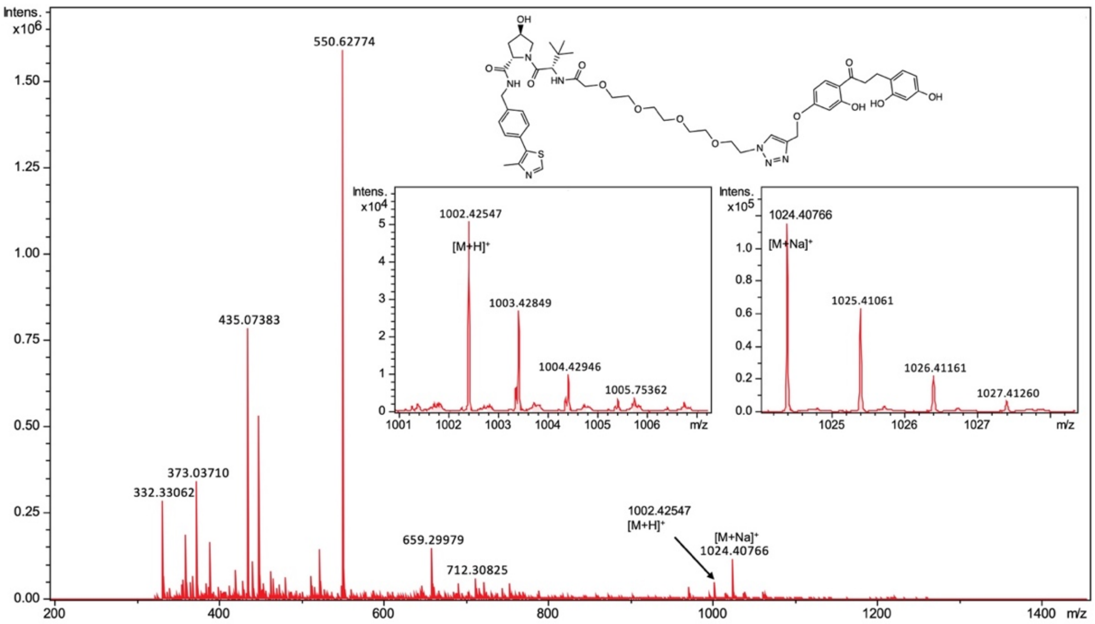

Supplementary Fig. 19. LC-MS analysis of DeTYR-3 in A375 cells.

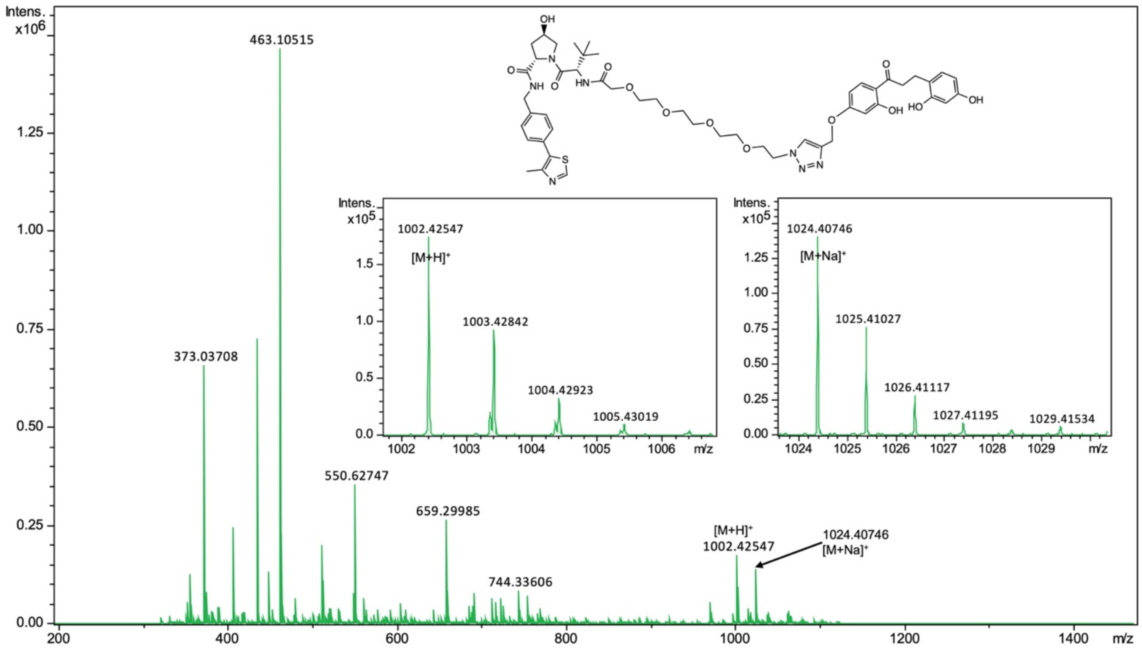

Supplementary Fig. 20. LC-MS analysis of DeTYR-3 in B16F10 cells.

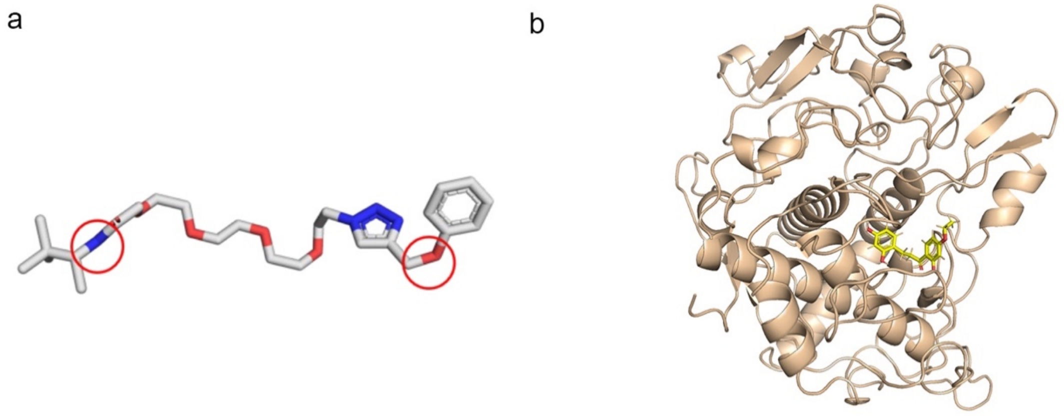

Supplementary Fig. 21. a, Structural conformation of the linker in DeTYR-3. Distance was measured by the two atoms (circled in red). **b**, mTYR (wheat)-Alk-TIn (yellow) complex showing in PathDock Protein-Protein docking.

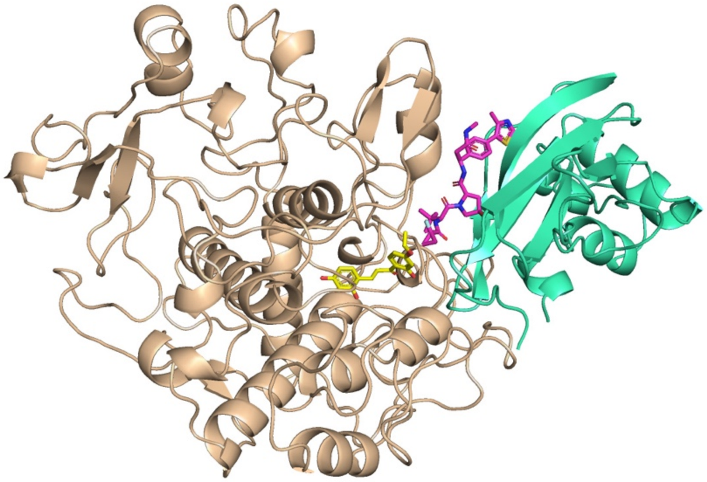

Supplementary Fig. 22. PathDock Protein-Protein docking of the bindings of mTYR (wheat), Alk-TIn (yellow), VHL (cyan), and VH032-Azi3 (magenta).

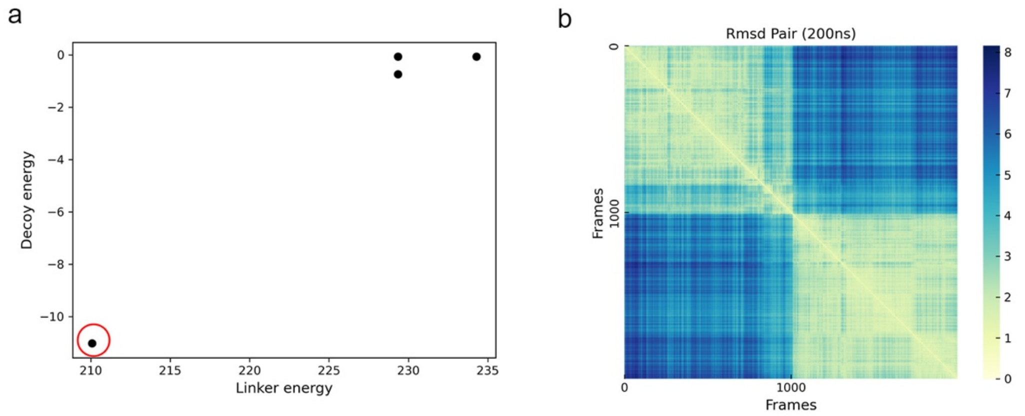

Supplementary Fig. 23. a, Ternary models score generated from Python script. The red circled model is chosen for the MD simulation. **b,** The CA atoms of the helix and all heavy atoms were used for alignment and RMSD calculation by the MDAnalysis Python package, respectively.

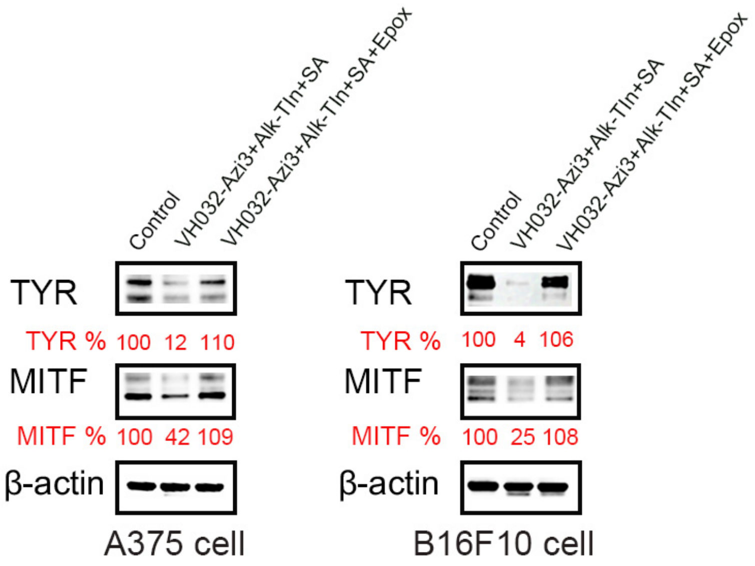

Supplementary Fig. 24. Western blot analysis of TYR and MITF in A375 and B16F10 cells treated with VH032-Azi3 (0.1 μM) + Alk-TIn (0.1 μM) + SA (0.5 μM) and VH032-Azi3 (0.1 μM) + Alk-TIn (0.1 μM) + SA (0.5 μM) + Epox (0.2 μM) for 24 h.

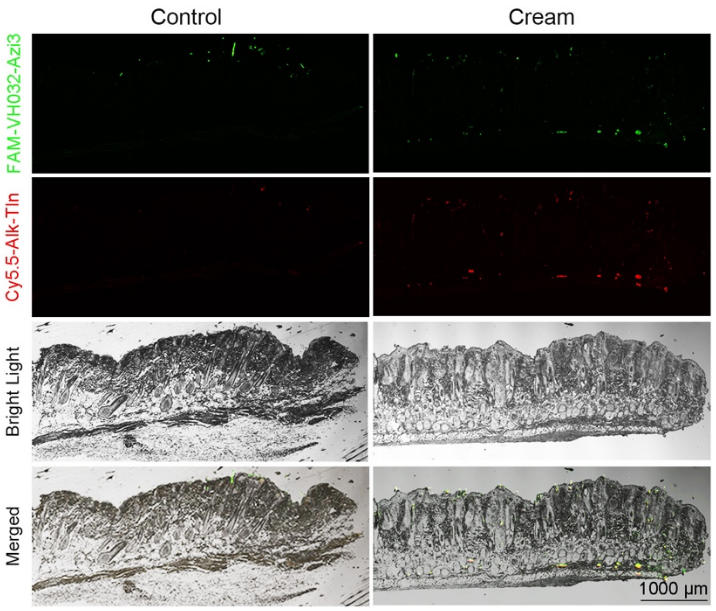

Supplementary Fig. 25. Representative confocal microscopy images of mouse skin tissues treated with FAM-VH032-Azi3 and Cy5.5-Alk-TIn in control and cream for 24 h, respectively. (n = 3 independent samples).

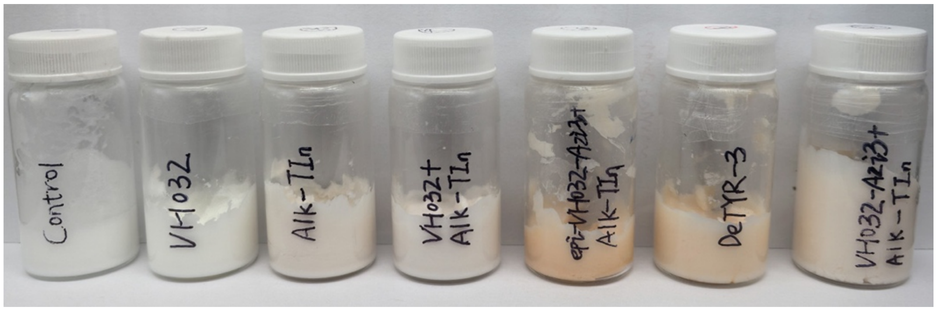

Supplementary Fig. 26. Images of seven different groups of skin creams. SA is added to VH032-Azi3 + Alk-TIn and epi-VH032-Azi3 + Alk-TIn groups.

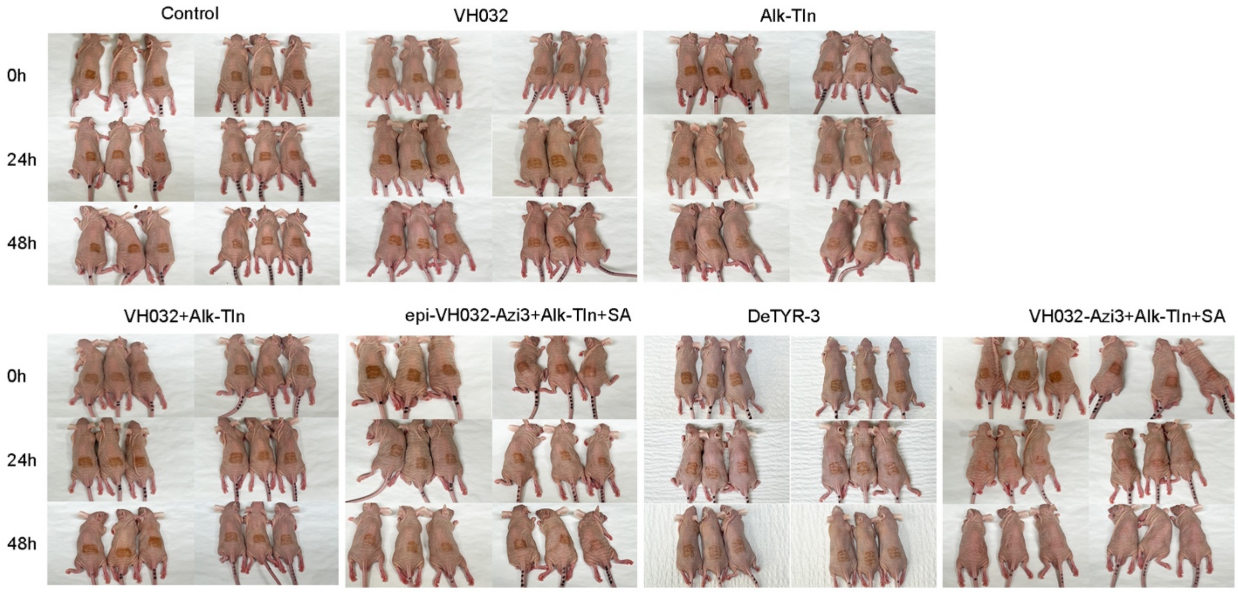

Supplementary Fig. 27. Images of the backs of mice treated with blank control, VH032, Alk-TIn, VH032 + Alk-TIn, epi-VH032-Azi3 + Alk-TIn + SA, DeTYR-3 and VH032-Azi3 + Alk-TIn + SA creams at different time points (n=6).

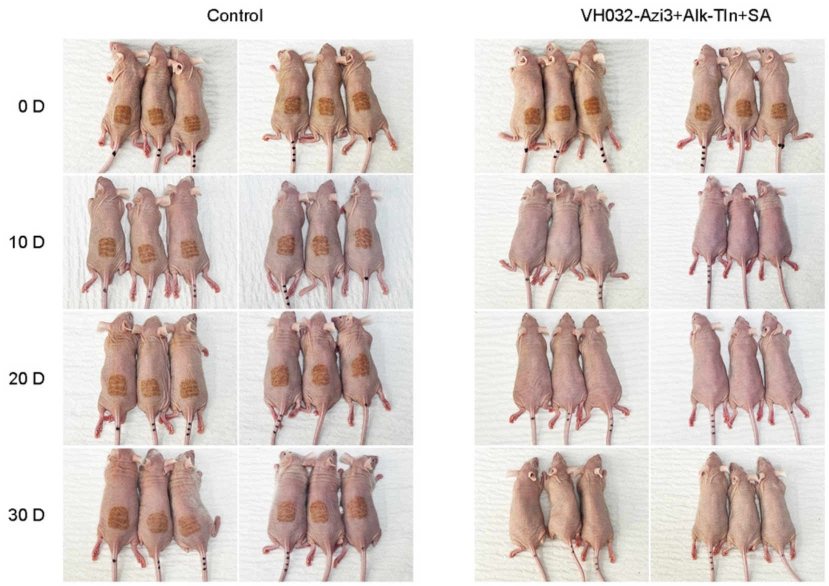

Supplementary Fig. 28. Images of square pigmentation patterns on the backs of mice treated with blank control and VH032-Azi3 + Alk-TIn + SA creams at different time points (n=6).

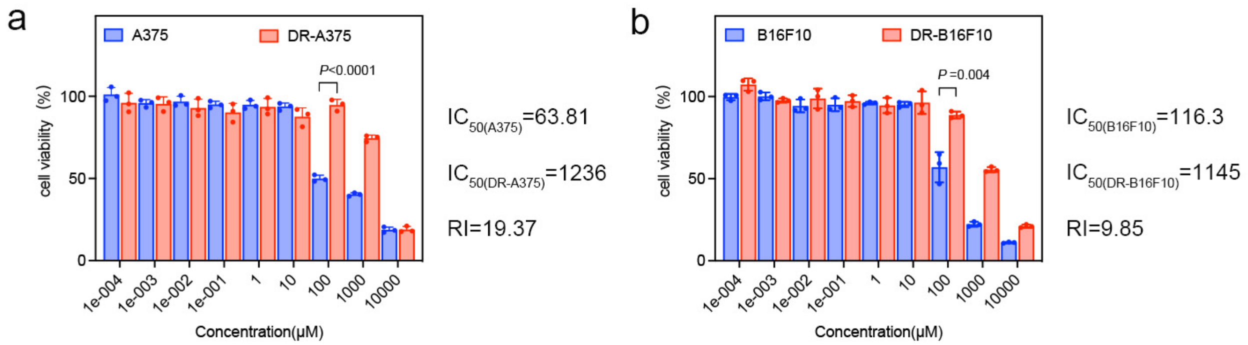

Supplementary Fig. 29. The resistance index (RI) of the DR-A375 (**a**) and DR-B16F10 (**b**) cells. RI > 5, which meets the requirements for drug-resistant cell lines. Statistical analysis was performed via unpaired Student's t-test (two-tailed).

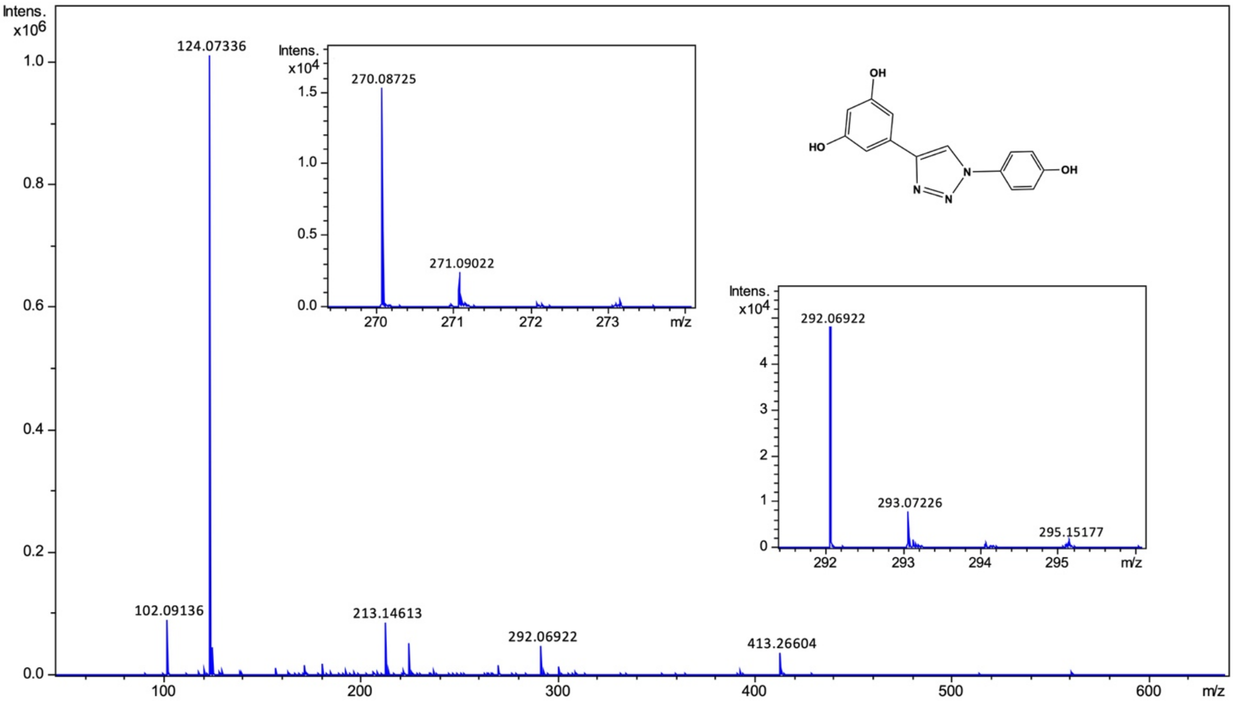

Supplementary Fig. 30. LC-MS analysis of Cd in DR-A375 cells.

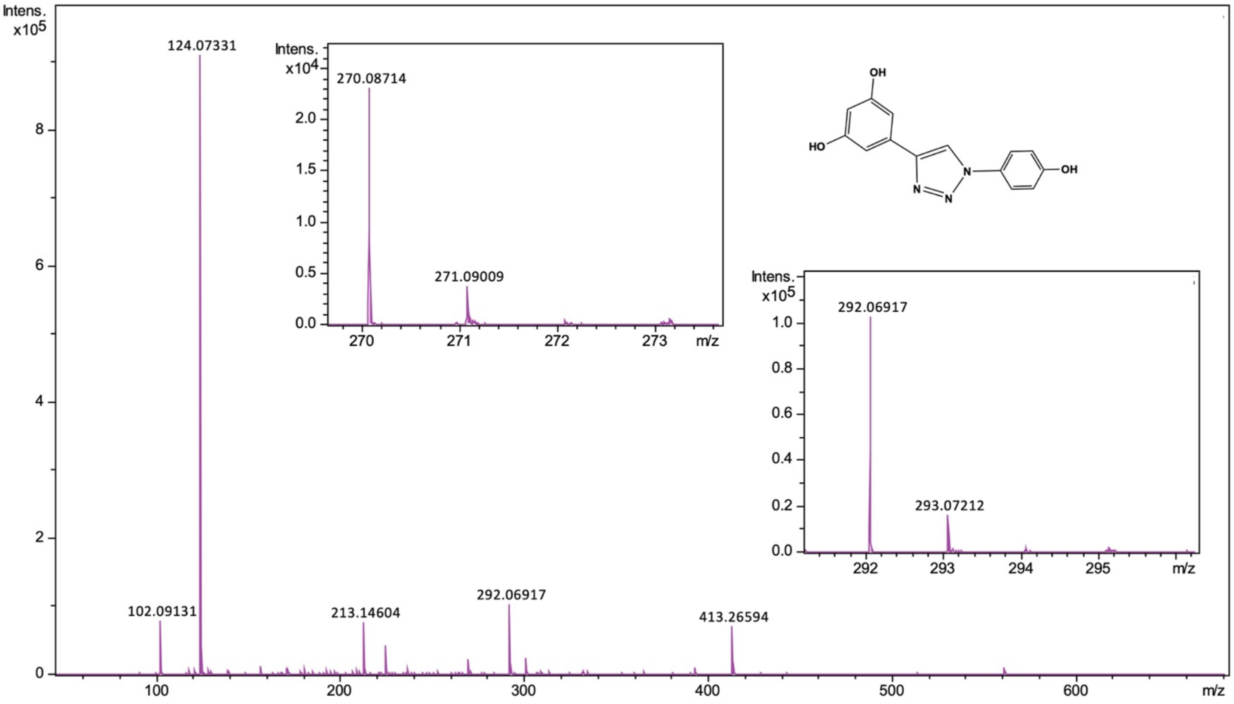

Supplementary Fig. 31. LC-MS analysis of Cd in DR-B16F10 cells.

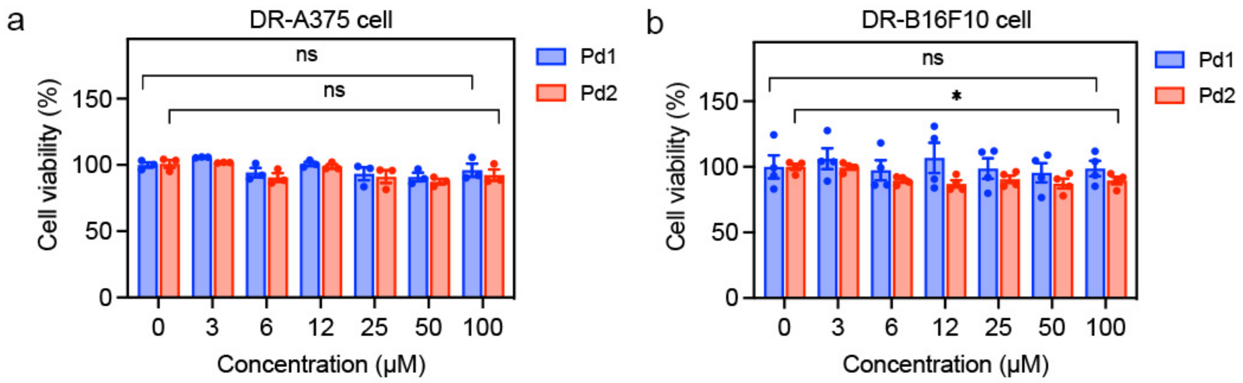

Supplementary Fig. 32. The viability of DR-A375 (**a**) and DR-B16F10 (**b**) cells treated with different concentrations of Pd1 and Pd2 through a CCK-8 assay. Data are presented as Mean ± standard error (SEM), (n = 4 biological replicates). Statistical analysis was performed via unpaired Student's t-test (two-tailed) (P=0.026, *P < 0.05).

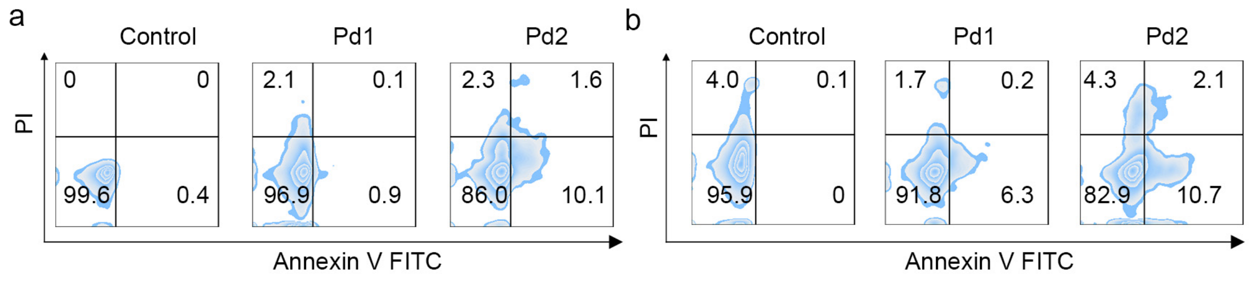

Supplementary Fig. 33. Flow cytometry analysis of cell apoptosis in DR-A375 (**a**) and DR-B16F10 (**b**) cells treated with Pd1 and Pd2 at 24 h.

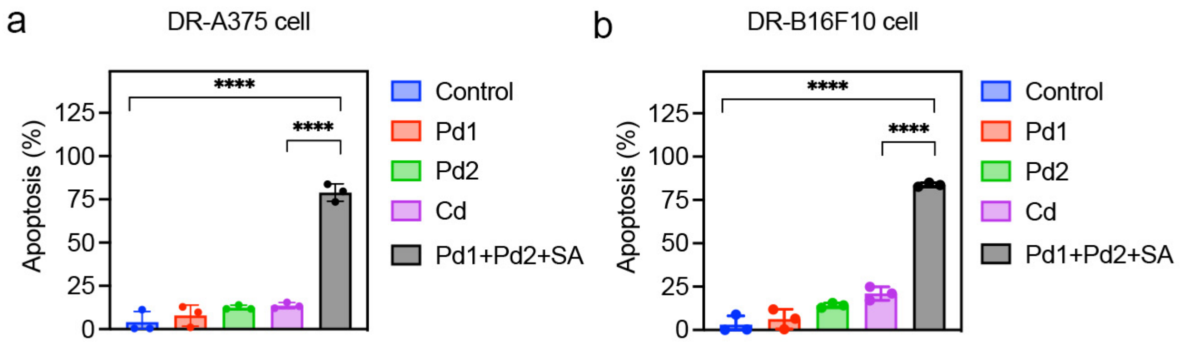

Supplementary Fig. 34. Quantitative analysis of apoptosis percentage in DR-A375 (**a**) and DR-B16F10 (**b**) cells treated with control, Pd1, Pd2, Cd and Pd1 + Pd2 + SA at 24 h. (n = 3 biological replicates). Statistical analysis was performed using ONE-WAY variance (ANOVA) by Dunnett's multiple comparisons (****P < 0.0001).

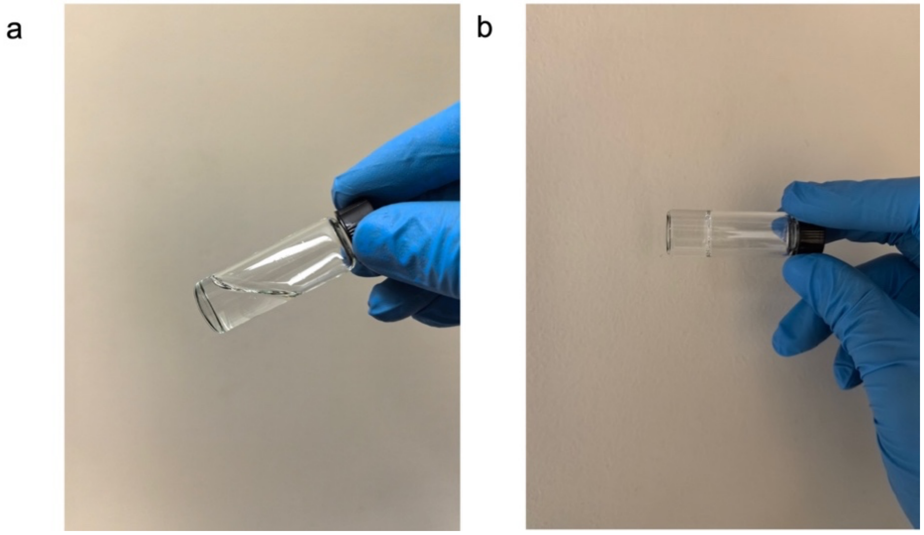

Supplementary Fig. 35. Gel formation of the Pluronic® F-127 solution at room temperature (**a**) and body temperature (**b**). (n = 3 independent experiments)

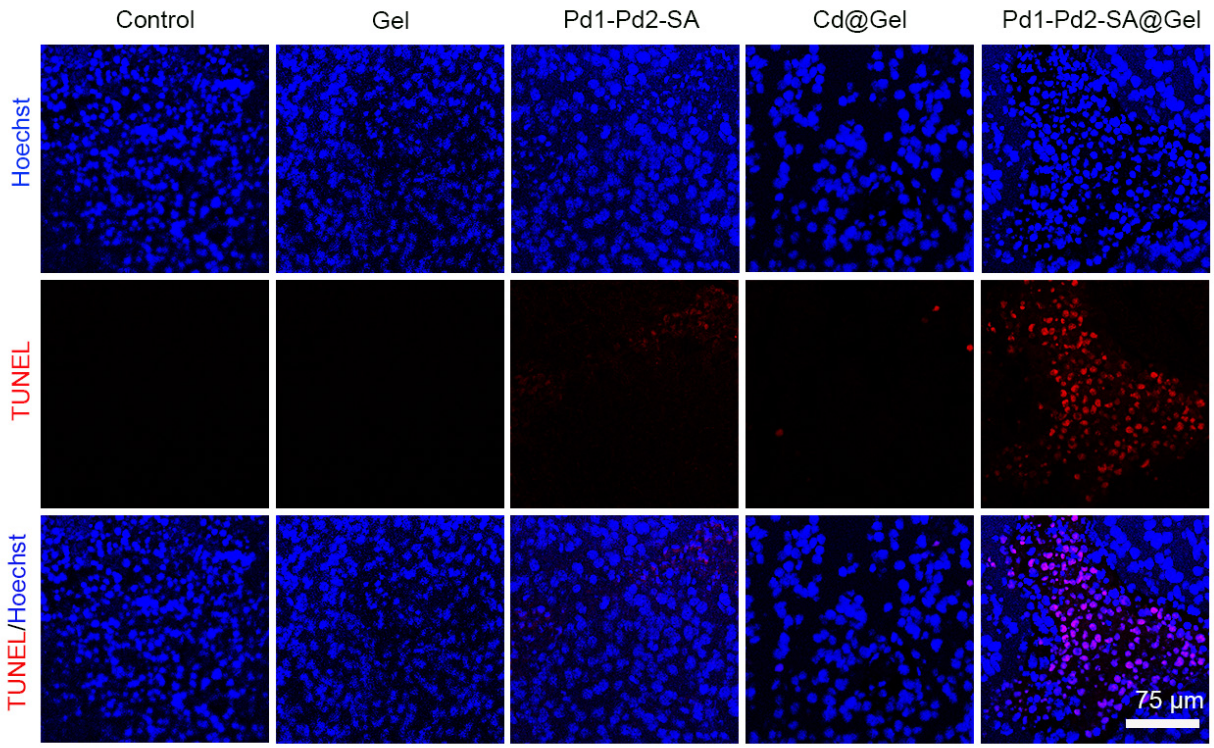

Supplementary Fig. 36. Representative TUNEL staining images of tumors after different treatments. n = three independent biological samples. (n = 3 independent samples)

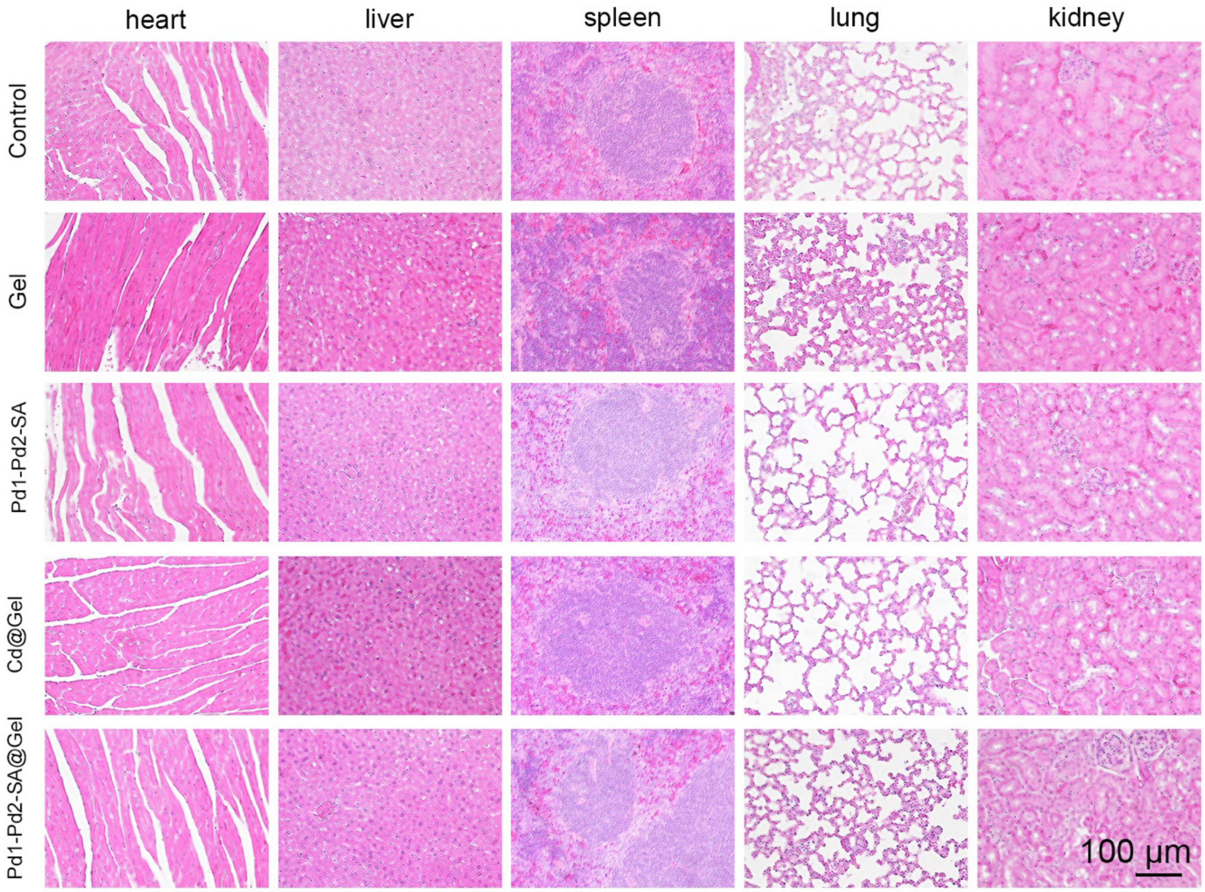

Supplementary Fig. 37. Representative H&E staining images of the major organs after different treatments. (n = 3 independent samples)

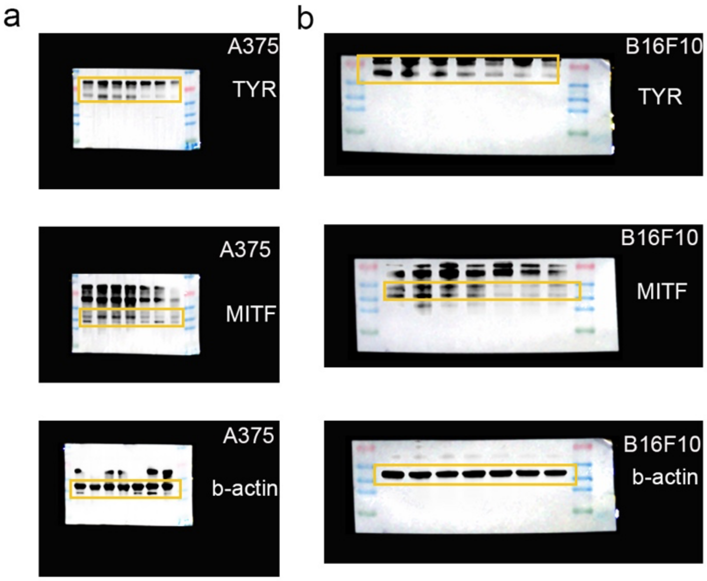

Supplementary Fig. 38. Uncropped scans of all blots and gels in Supplementary Fig. 13.

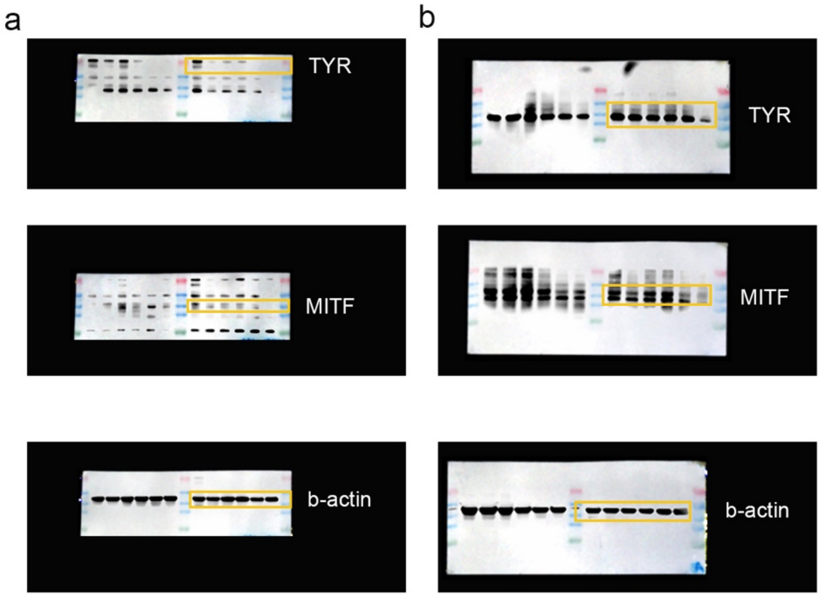

Supplementary Fig. 39. Uncropped scans of all blots and gels in Supplementary Fig. 16.

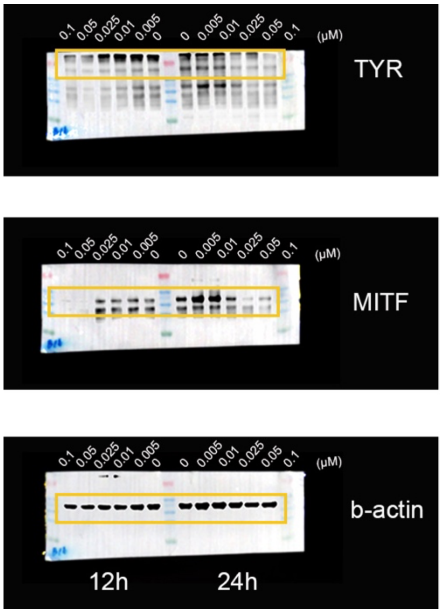

Supplementary Fig. 40. Uncropped scans of all blots and gels in Supplementary Fig. 17.

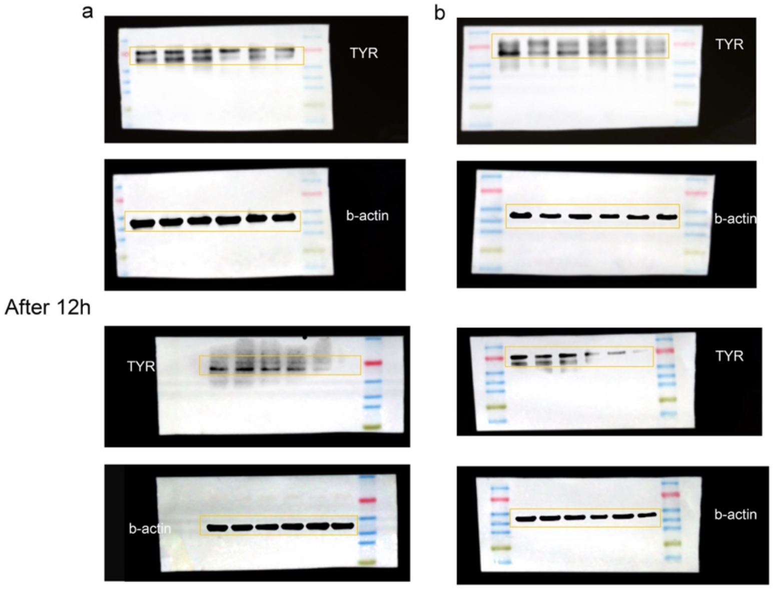

Supplementary Fig. 41. Uncropped scans of all blots and gels in Supplementary Fig. 18.

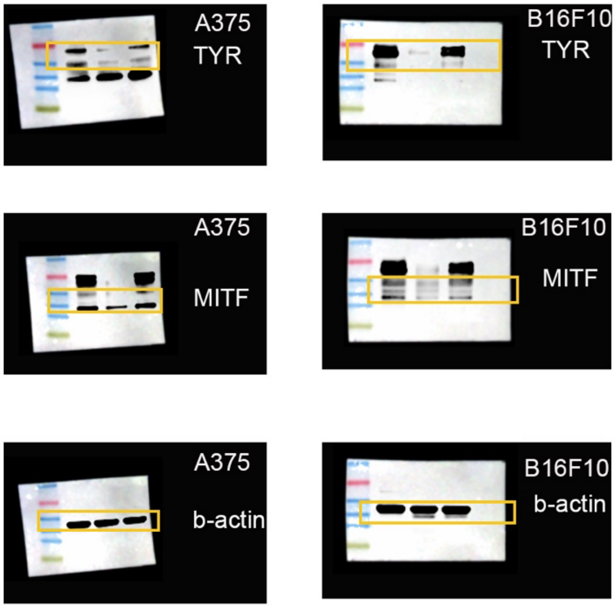

Supplementary Fig. 42. Uncropped scans of all blots and gels in Supplementary Fig. 24.

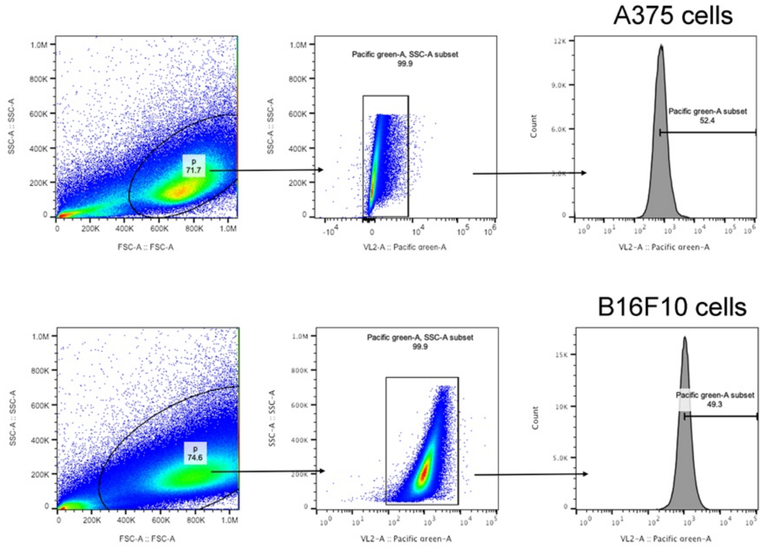

Supplementary Fig. 43. Gating strategy for flow cytometry analysis of Fig. 1f and Fig. 1g.

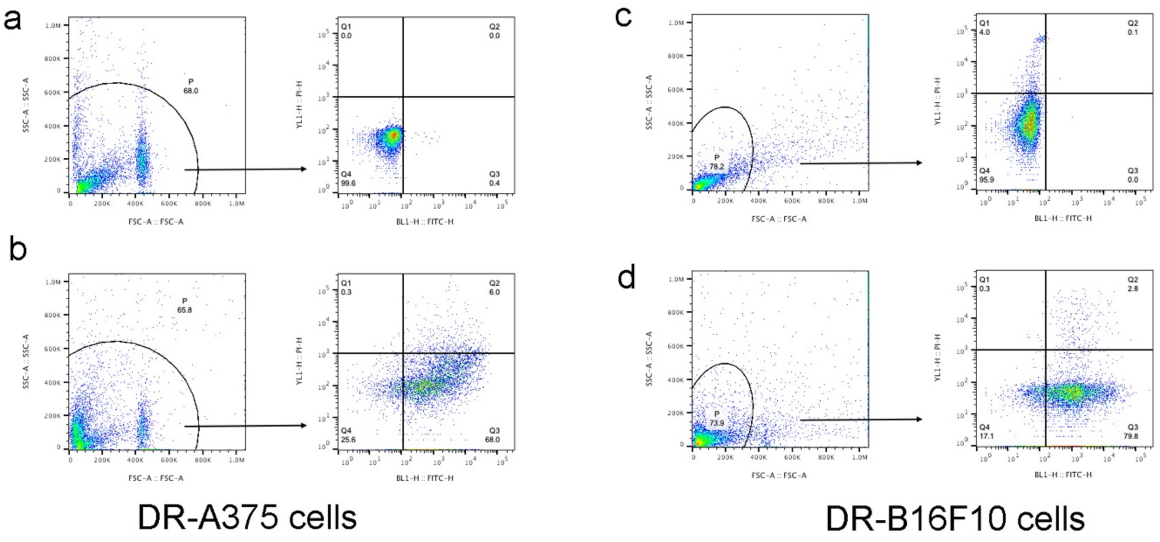

Supplementary Fig. 44. Gating strategy for flow cytometry analysis of Fig. 5d and Supplementary Fig. 33.

Synthesis of Chemical Molecules

3-(2,4-dihydroxyphenyl)-1-(2-hydroxy-4-(prop-2-yn-1-yloxy)phenyl)propan-1-one (Alk-TIn)

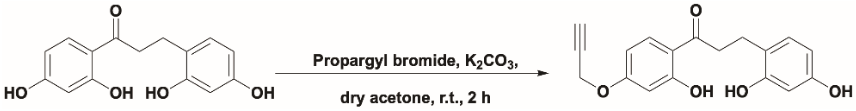

1,3-Bis (2,4-dihydroxy phenyl) propane-1-one (0.1 mmol) was added to dry acetone (2 mL) solution, stirred at room temperature for 5 min. Afterward, K_2CO_3 (0.2 mmol) and propargyl bromide (0.15 mmol) were added[13]. The reaction solution was further stirred for 2 h, and the reaction progress was monitored by thin layer chromatography. Once the reaction was complete, the reaction mixture was diluted with brine water and extracted thrice with ethyl acetate. Finally, the combined extract was passed through Na_2SO_4, and the solvent was evaporated under vacuum. The reaction was monitored by TLC, and the crude product was subjected to column chromatography (Hexane: EtOAc) to give pure compound **1**. Yield: 89%.

^{1}H NMR (400 MHz, DMSO-d_6) δ 12.66 (s, 1 H), 9.23 (s, 1 H), 8.98 (s, 1 H), 7.9 (d, 1 H), 6.85 (d, 1 H), 6.54 (d, 2 H), 6.28 (d, 1 H), 6.12 (dd, 2 H), 4.89 (d, 2 H), 3.63 (t, 1 H), 3.16 (m, 2 H), 2.75 (m, 2 H).

^{13}C NMR (400 MHz, DMSO-d_6) δ 205.07, 163.83, 163.27, 156.60, 155.83, 132.58, 130.10, 117.21, 113.87, 107.60, 105.94, 102.39, 101.95, 78.85, 78.51, 55.85, 38.46, 24.77.

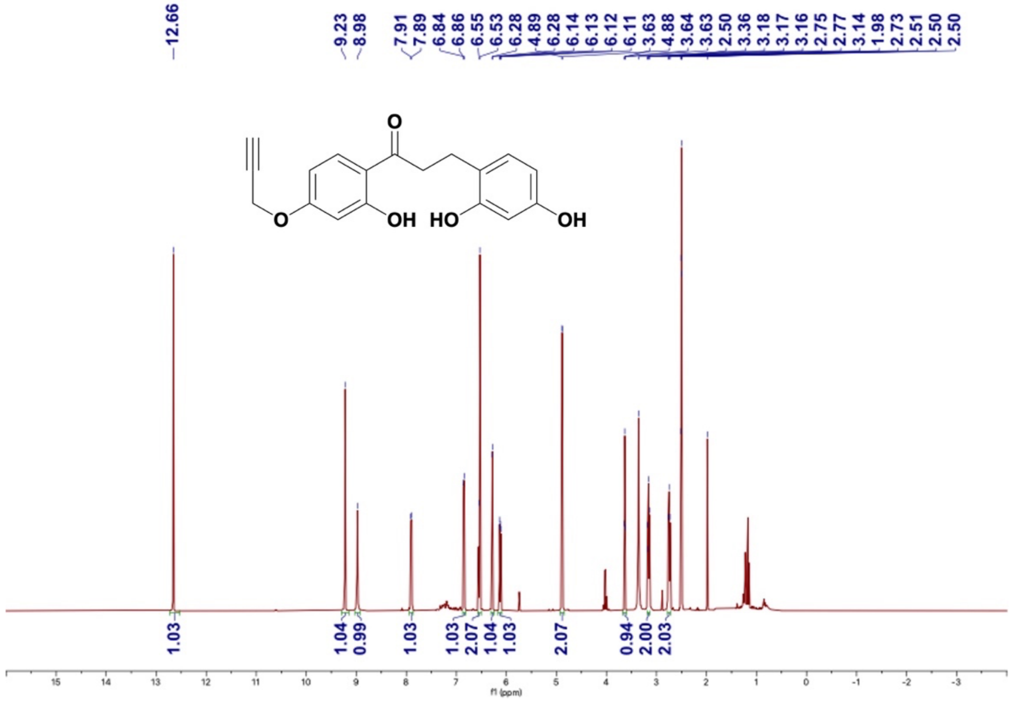

Supplementary Fig. 45. ^{1}H NMR spectrum of 3-(2,4-dihydroxyphenyl)-1-(2-hydroxy-4-(prop-2-yn-1-yloxy) phenyl) propan-1-one, **Alk-TIn**. (DMSO-d_6, δ = 2.5).

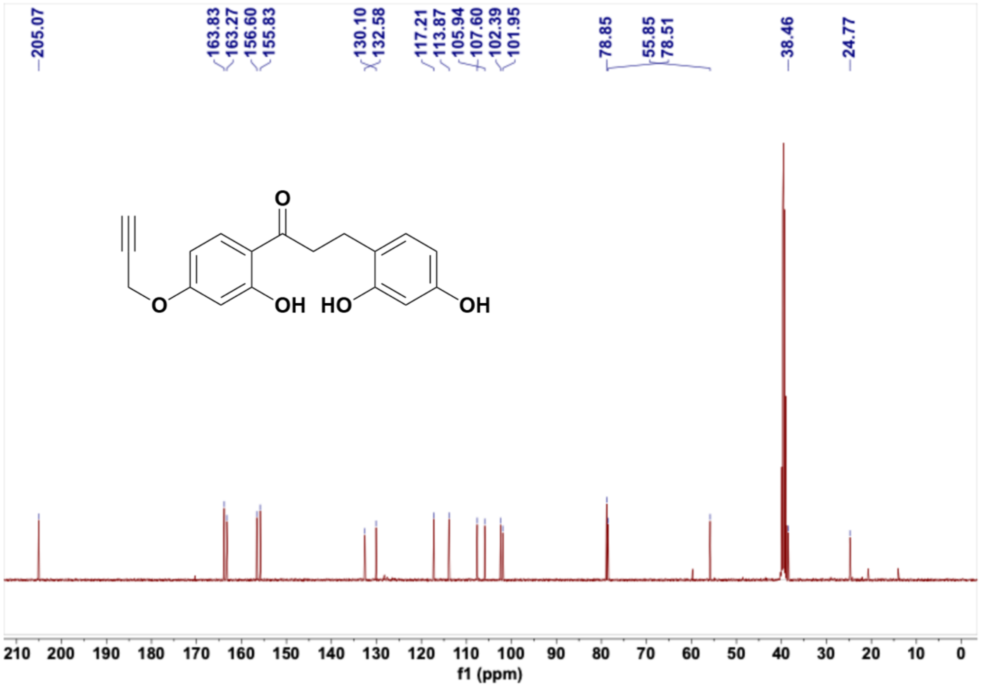

Supplementary Fig. 46. ^{13}C NMR spectrum of 3-(2,4-dihydroxyphenyl)-1-(2-hydroxy-4-(prop-2-yn-1-yloxy) phenyl) propan-1-one, **Alk-TIn**. (DMSO-d_6, δ = 38.5).

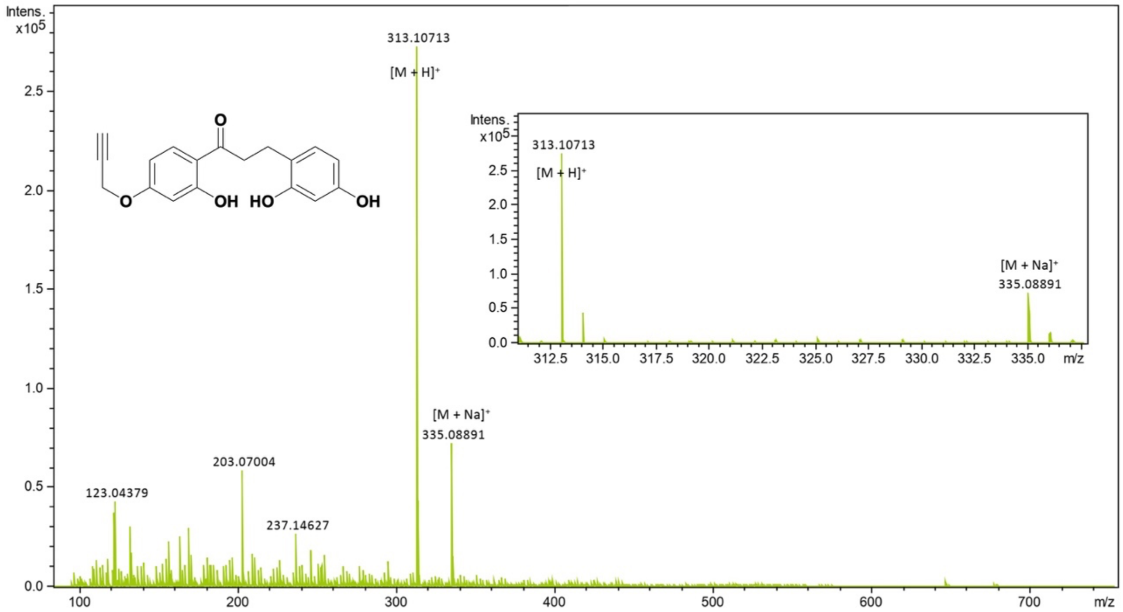

Supplementary Fig. 47. LC-MS spectrum of 3-(2,4-dihydroxyphenyl)-1-(2-hydroxy-4-(prop-2-yn-1-yloxy) phenyl) propane-1-one, **Alk-TIn**.

tert-butyl (2S, 4S)-4-hydroxy-2-((4-(4-methylthiazol-5-yl) benzyl) carbamoyl) pyrrolidine-1-carboxylate (1)

(4-(4-methylthiazol-5-yl)phenyl)methanamine1 (0.243 mmol, 1 eq.) was dissolved in dichloromethane (DCM), then (2S, 4S)-1-(tert-butoxycarbonyl)-4-hydroxypyrrolidine-2-carboxylic acid (0.243 mmol, 1 eq.) and 1-[Bis (dimethylamino) methylene]-1H-1,2,3-triazolo[4,5-b]pyridinium 3-oxid hexafluorophosphate (HATU) (0.268 mmol, 1.1 eq.) were added. After the pH of the reaction was adjusted to > 9 by the addition of *N*, *N*-Diisopropylethyl amine (0.972 mmol, 4 eq.), the reaction was stirred for 2 h at 25 °C. The reaction mixture was then washed with water, and the organic phase was dried over Na_2SO_4. After removing the solvent in vacuum, the residue was purified by flash column chromatography using a gradient of 10% to 70% Acetone in Hexane[14]. Yield: 60%.

^{1}H-NMR (400 MHz, *d*-$CDCl_3$) δ 1.45 (s, 9 H), 2.18 (m, 1H), 2.35-2.38 (m, 1 H), 2.52 (s, 3 H), 3.51 (m, 2 H), 4.41 (m, 4 H), 4.56 (dd, 1 H, J(H, H)=7.1 Hz, J(H, H)= 14.9 Hz), 7.32-7.39 (m, 4 H), 7.59 (m, 1 H), 8.68 (s, 1 H).

^{13}C NMR (400 MHz, *d*-$CDCl_3$) δ173.91, 155.68, 150.80, 148.30, 137.84, 131.89, 129.58, 127.80, 81.10, 70.80, 59.80, 56.88, 55.85, 55.04, 43.36, 38.63, 36.61, 29.75, 28.35, 15.78, 12.54.

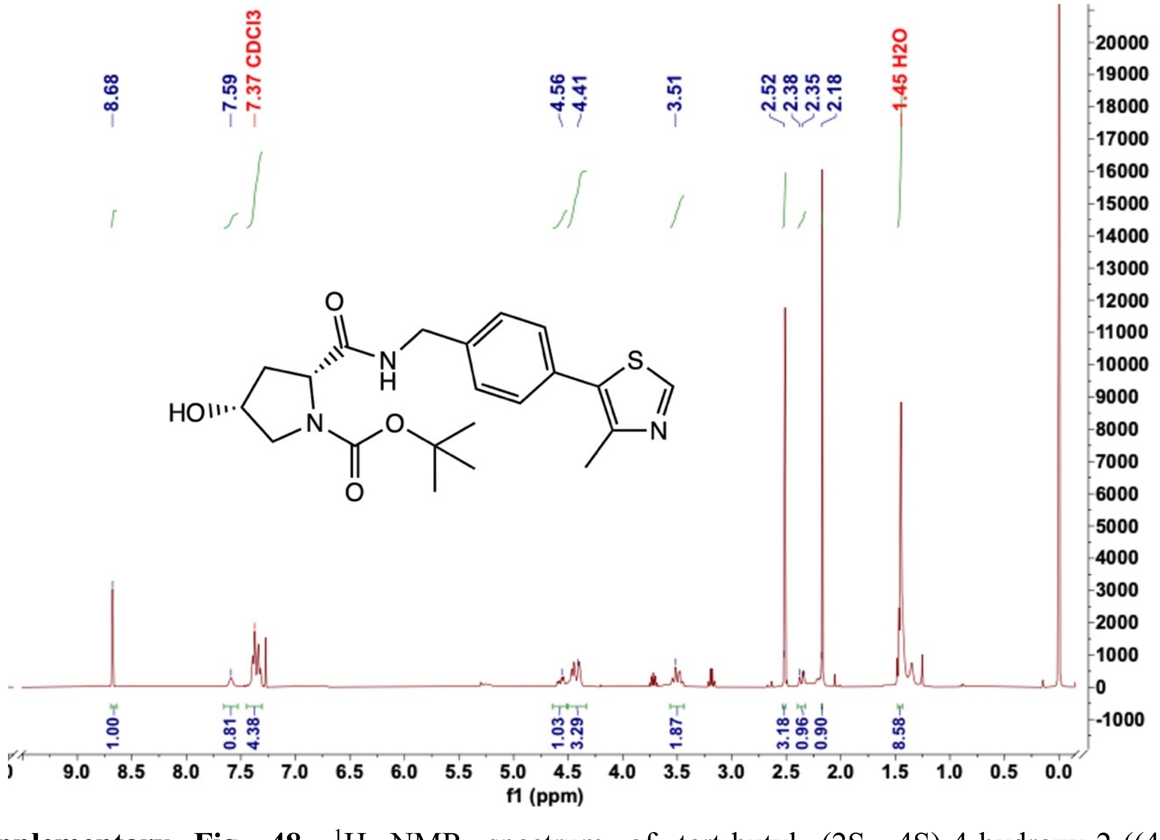

Supplementary Fig. 48. ^{1}H NMR spectrum of tert-butyl (2S, 4S)-4-hydroxy-2-((4-(4-methylthiazol-5-yl) benzyl) carbamoyl) pyrrolidine-1-carboxylate, **1**. (*d*-$CDCl_3$).

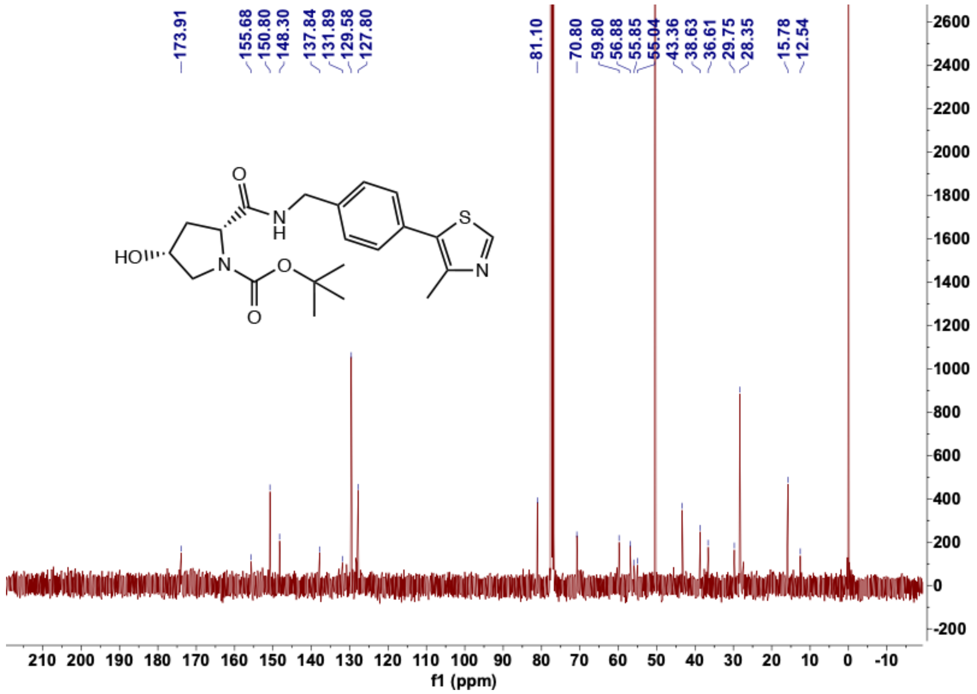

Supplementary Fig. 49. ^{13}C NMR spectrum of tert-butyl (2S, 4S)-4-hydroxy-2-((4-(4-methylthiazol-5-yl) benzyl) carbamoyl) pyrrolidine-1-carboxylate, **1**. (*d*-CDCl3).

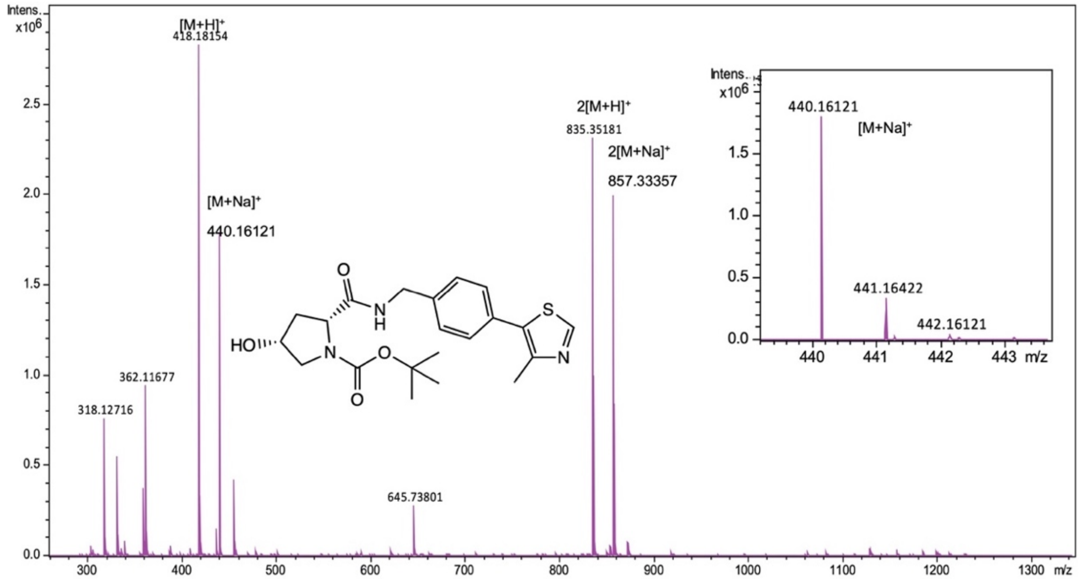

Supplementary Fig. 50. LC-MS spectrum of tert-butyl (2S, 4S)-4-hydroxy-2-((4-(4-methylthiazol-5-yl) benzyl) carbamoyl) pyrrolidine-1-carboxylate, **1**.

General procedure for Boc-deprotection:

The *N*-Boc-protected compound was dissolved in dichloromethane (10 mL/1 mmol). Trifluoroacetic acid (10 mL/1 mmol) was further added, and the reaction mixture was stirred at room temperature for 2 h. The solvent was removed under reduced pressure. The dichloromethane

(5 mL/1 mmol) was added three times, and then the solvent was again removed in a vacuum to remove residual trifluoroacetic acid.

tert-butyl ((S)-1-((2S, 4S)-4-hydroxy-2-((4-(4-methylthiazol-5-yl) benzyl) carbamoyl) pyrrolidin-1-yl)-3,3-dimethyl-1-oxobutan-2-yl) carbamate (2)

1 was boc-deprotected as described below to obtain **1 * ** TFA. **1 * ** TFA (0.14 mmol, 1 eq.) and (S)-2-((tert-butoxycarbonyl) amino)-3,3-dimethylbutanoic acid (0.14 mmol, 1 eq.) were dissolved in DCM (10 mL). After the addition of HATU (0.21 mmol, 1.5 eq.), the pH was adjusted to > 9 by the addition of *N*, *N*-Diisopropylethyl amine (0.56 mmol, 4 eq.), and the reaction was stirred at 25 °C for 2 h. The reaction mixture was then washed with water, and the remaining organic phase was dried over magnesium sulfate. After removing the solvent in a vacuum, the residue was purified by flash column chromatography using a gradient of 10% to 60% Acetone in Hexane[14]. Yield: 50%.

^{1}H NMR (400 MHz, *d*-$CDCl_3$) δ 0.92 (s, 7 H), 1.41 (s, 10 H), 2.16-2.22 (m, 3 H), 2.30 (m, 1 H), 2.51 (s, 3 H), 3.88-3.84(m, 2 H), 4.21 (d, 1 H, J(H, H)= 8.4 Hz), 4.29 (dd, 1 H, J(H, H)= 5.1 Hz, J(H, H)= 14.9 Hz), 4.48 (s, 1 H), 4.64 (dd, 1 H, J(H, H)= 7.1 Hz, J(H, H)= 14.9 Hz), 4.77 (d, 1 H, J(H, H)= 8.8 Hz), 5.12 (d, 1H, J(H, H)= 9.0 Hz), 5.31 (s, 1 H), 7.33-7.39 (m, 4 H), 8.01 (m, 1 H), 8.71 (s, 1 H).

^{13}C NMR (400 MHz, *d*-$CDCl_3$) δ172.74, 162.78, 155.69, 150.58, 148.30, 138.09, 137.51, 131.65, 131.00, 129.56, 128.08, 127.96, 79.83, 77.42, 77.10, 76.79, 71.09, 59.87, 58.43, 43.43, 36.57, 28.33, 26.57, 26.24, 15.93.

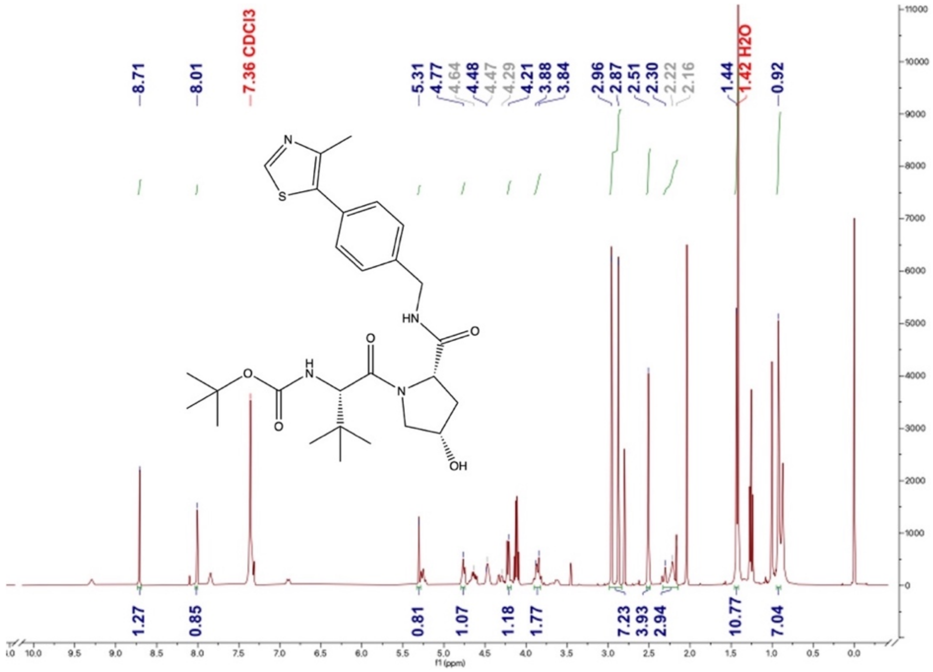

Supplementary Fig. 51. ^{1}H NMR spectrum of tert-butyl ((S)-1-((2S, 4S)-4-hydroxy-2-((4-(4-methylthiazol-5-yl) benzyl) carbamoyl) pyrrolidin-1-yl)-3,3-dimethyl-1-oxobutan-2-yl) carbamate **2**. (*d*-$CDCl_3$).

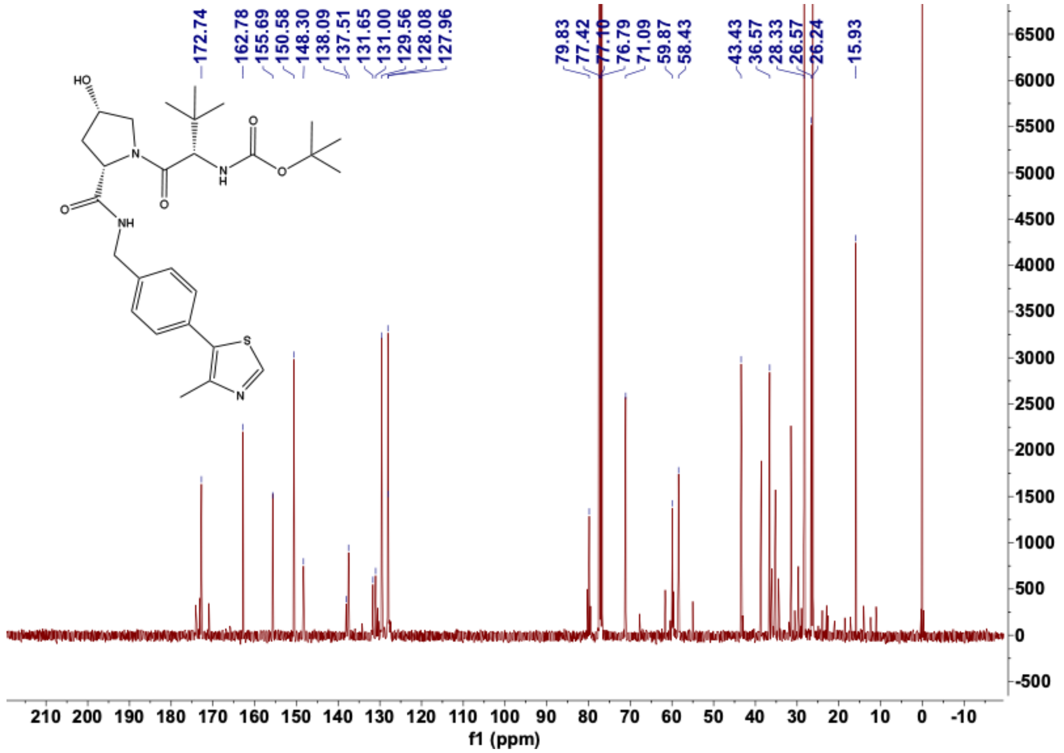

Supplementary Fig. 52. ^{13}C NMR spectrum of tert-butyl ((S)-1-((2S, 4S)-4-hydroxy-2-((4-(4-methylthiazol-5-yl) benzyl) carbamoyl) pyrrolidin-1-yl)-3,3-dimethyl-1-oxobutan-2-yl) carbamate **2**. (*d*-$CDCl_3$).

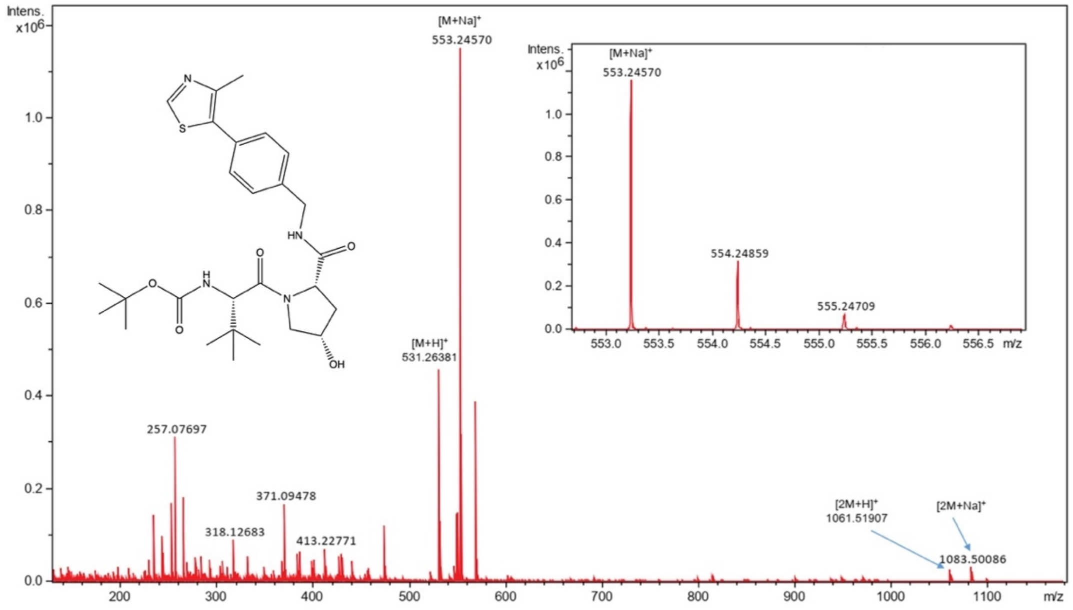

Supplementary Fig. 53. LC-MS spectrum of tert-butyl ((S)-1-((2S, 4S)-4-hydroxy-2-((4-(4-methylthiazol-5-yl) benzyl) carbamoyl) pyrrolidin-1-yl)-3,3-dimethyl-1-oxobutan-2-yl) carbamate **2**.

(2S, 4S)-1-((S)-17-azido-2-(tert-butyl)-4-oxo-6,9,12,15-tetraoxa-3-azaheptadecanoyl)-4-hydroxy-N-(4-(4-methylthiazol-5-yl) benzyl) pyrrolidine-2-carboxamide (epi-VH032-Azi3)

2 was boc-deprotected as described below to obtain **2** * TFA. The **2** * TFA (0.1 mmol, 1 eq.) was added to a solution of 14-azido-3,6,9,12-tetraoxatetradecanoic acid (0.12 mmol, 1.2 eq.) in DCM (4 mL). HATU (0.15 mmol, 1.5 eq.) was added, and the pH was adjusted to > 9 by the addition of DIPEA (0.4 mmol, 4 eq.). After stirring for 4 h at 25 °C, the reaction mixture was extracted with water. The organic phase was dried over magnesium sulfate and then evaporated to dryness. The crude product was purified by flash column chromatography using a gradient of 0%-6% of methanol in dichloromethane[14]. Yield: 40%.

^{1}H NMR (400 MHz, *d*-$CDCl_3$) δ 0.93 (s, 9 H), 2.19-2.21 (m, 1 H), 2.33-2.36 (s, 1 H), 2.51 (s, 3 H), 3.25 (t, 2 H, J(H, H)= 5.1 Hz), 3.64-3.67 (m, 14 H), 3.79-3.81 (m, 1 H), 3.89-3.93 (m, 1 H), 3.99-4.06 (m, 2 H), 4.28-4.30 (dd, 1 H, J(H, H)= 5.1 Hz, J(H, H)= 14.9 Hz), 4.46-4.48 (m, 1 H),4.52-4.55 (d, 1 H, J(H, H)= 9.2 Hz), 4.60-4.65 (dd, 1 H, J(H, H)= 7.1 Hz, J(H, H)= 14.9 Hz), 4.71-4.73 (d, 1 H, J(H, H)= 9.0 Hz), 5.53-5.56 (d, 1 H, J(H, H)= 9.9 Hz), 7.11-7.14 (d, 1 H, J(H, H)= 9.1 Hz), 7.33-7.39 (m, 4 H), 7.52-7.53(m, 1 H), 8.68 (s, 1 H).

^{13}C NMR (400 MHz, *d*-$CDCl_3$) δ173.38, 171.45, 170.62, 151.11, 137.97, 132.14, 129.66, 128.11, 106.13, 71.25, 71.16, 70.72, 70.69, 70.66, 70.47, 70.35, 70.15, 69.89, 60.08, 58.12, 56.94, 54.88, 35.44, 29.83, 26.38, 15,75.

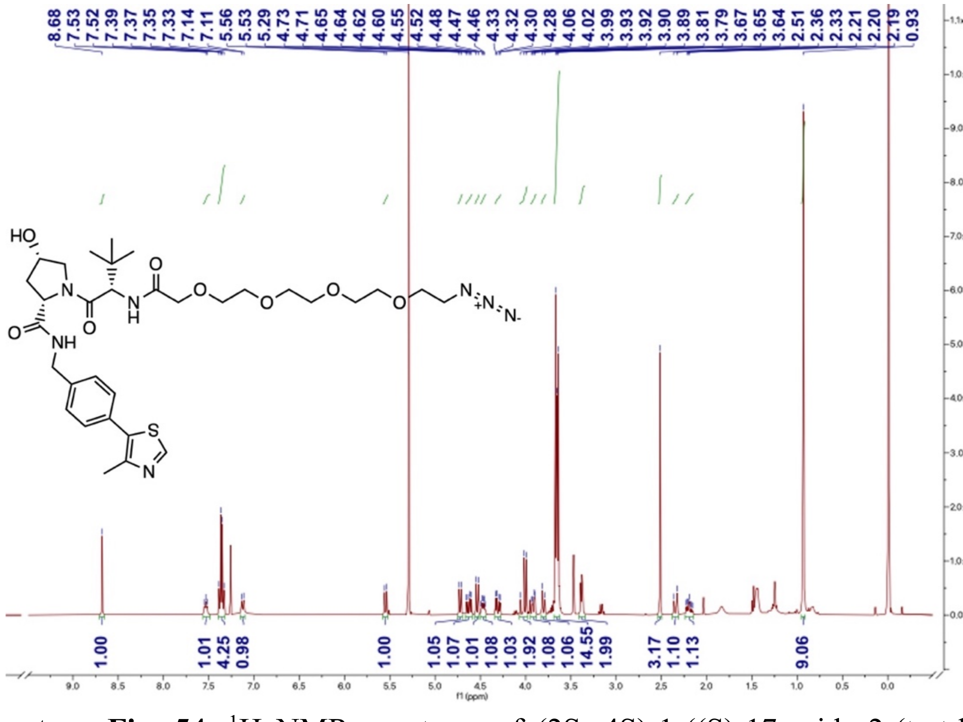

Supplementary Fig. 54. ^{1}H NMR spectrum of (2S, 4S)-1-((S)-17-azido-2-(tert-butyl)-4-oxo-6,9,12,15-tetraoxa-3-azaheptadecanoyl)-4-hydroxy-N-(4-(4-methylthiazol-5-yl) benzyl)pyrrolidine-2-carboxamide, **epi-VH032-Azi3**.(*d*-$CDCl_3$).

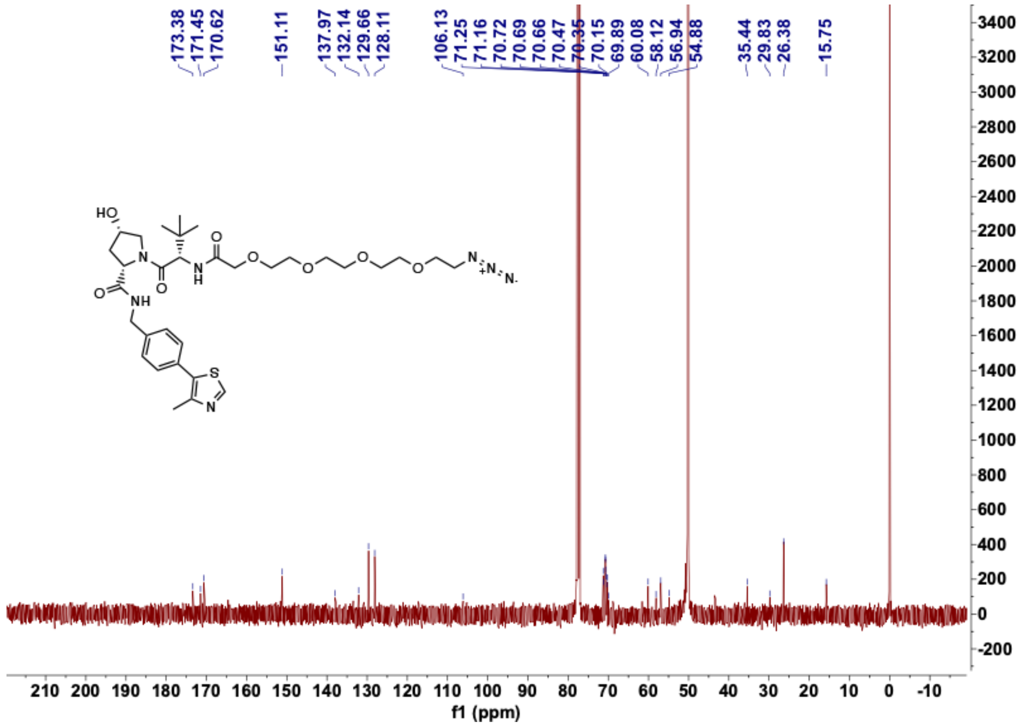

Supplementary Fig. 55. ^{13}C NMR spectrum of (2S, 4S)-1-((S)-17-azido-2-(tert-butyl)-4-oxo-6,9,12,15-tetraoxa-3-azaheptadecanoyl)-4-hydroxy-N-(4-(4-methylthiazol-5-yl)benzyl) pyrrolidine-2-carboxamide, **epi-VH032-Azi3**. (*d*-$CDCl_3$).

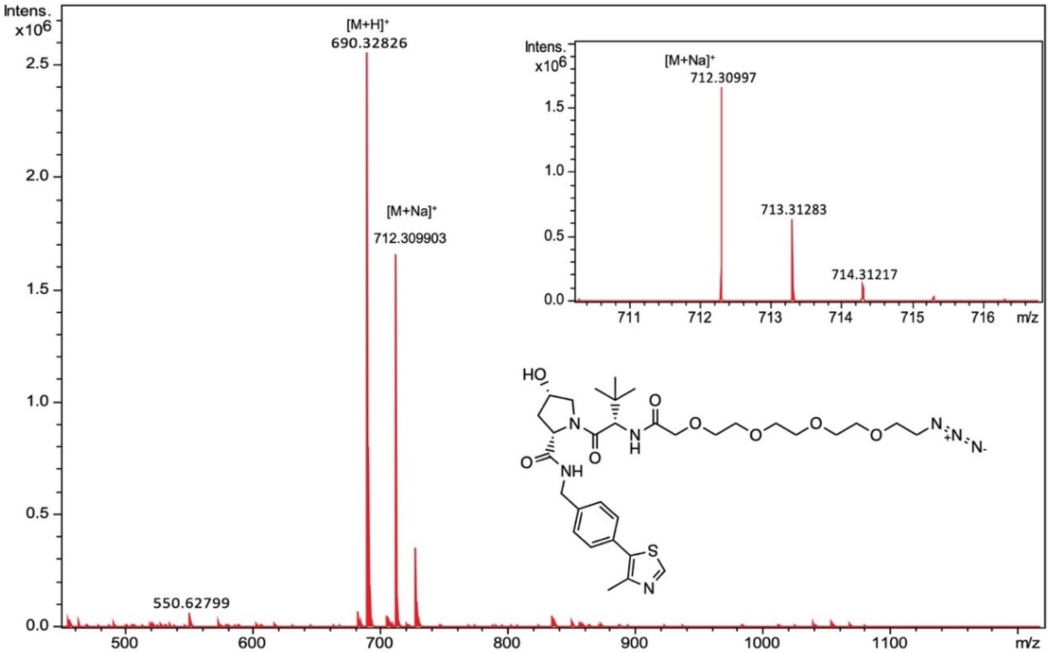

Supplementary Fig. 56. LC-MS spectrum of (2S, 4S)-1-((S)-17-azido-2-(tert-butyl)-4-oxo-6,9,12,15-tetraoxa-3-azaheptadecanoyl)-4-hydroxy-N-(4-(4-methylthiazol-5-yl)benzyl)pyrrolidine-2-carboxamide, **epi-VH032-Azi3**.

5-azidobenzene-1,3-diol (Pd1)
Phloroglucinol (7.95 mmol) and 7.8 mL $NH_3 \cdot H_2O$ were mixed under a N_2 atmosphere. The mixture was stirred for 24 h, then the solution was distilled in a vacuum to remove the solvent. HCl (6 M) was added under an ice bath to produce the hydrochloric acid salt. The solvent was distilled in a vacuum, and then the product was purified through MeOH/CH_2Cl_2 reprecipitation. The obtained yellow precipitation is the precursor of **Pd1**[15].

0.5 g of 5-Aminoresorcinol hydrochloride was added to a solution of 1.25 mL distilled water and 1.25 mL conc. HCl at 0 °C. $NaNO_2$ (2.8 mmol) dissolved in 1.25 mL distilled water was added to the above solution slowly (> 5 min). After 10 min, NaN_3 (3.3 mmol) dissolved in 1.25 mL distilled water was added, and the reaction was then allowed to stir for an additional 40 min at 0 °C. The resulting solution was extracted with EtOAc (3 × 20 mL). Organic layers were combined, washed with brine (1 × 20 mL), dried over anhydrous Na_2SO_4, and concentrated under a vacuum. The crude product was chromatographed with Hexane/EtOAc as eluent, to afford the light-yellow crystal, **Pd1**. Yield: 80%.
^{1}H NMR (400 MHz, *d*-acetone) δ 8.57 (s, 1 H), 6.21 (s, 1 H), 6.08 (d, J = 2.1 Hz, 1 H).
^{13}C NMR (400 MHz, *d*-acetone) δ 159.63, 141.61, 99.79, 97.71.

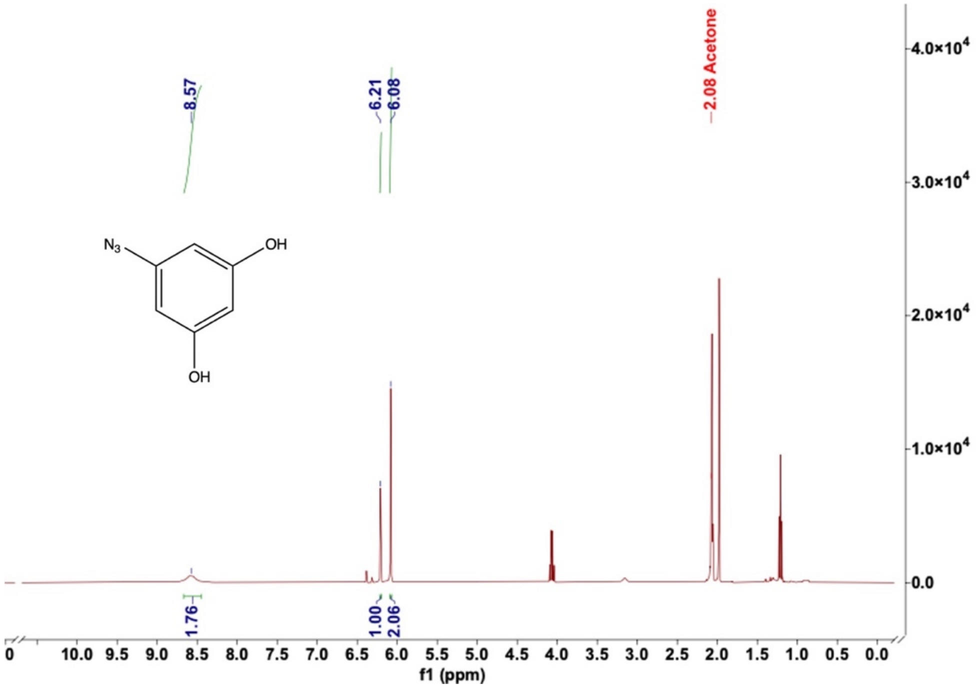

Supplementary Fig. 57. ^{1}H NMR spectrum of 5-azidobenzene-1, 3-diol, **Pd1** (*d*-acetone).

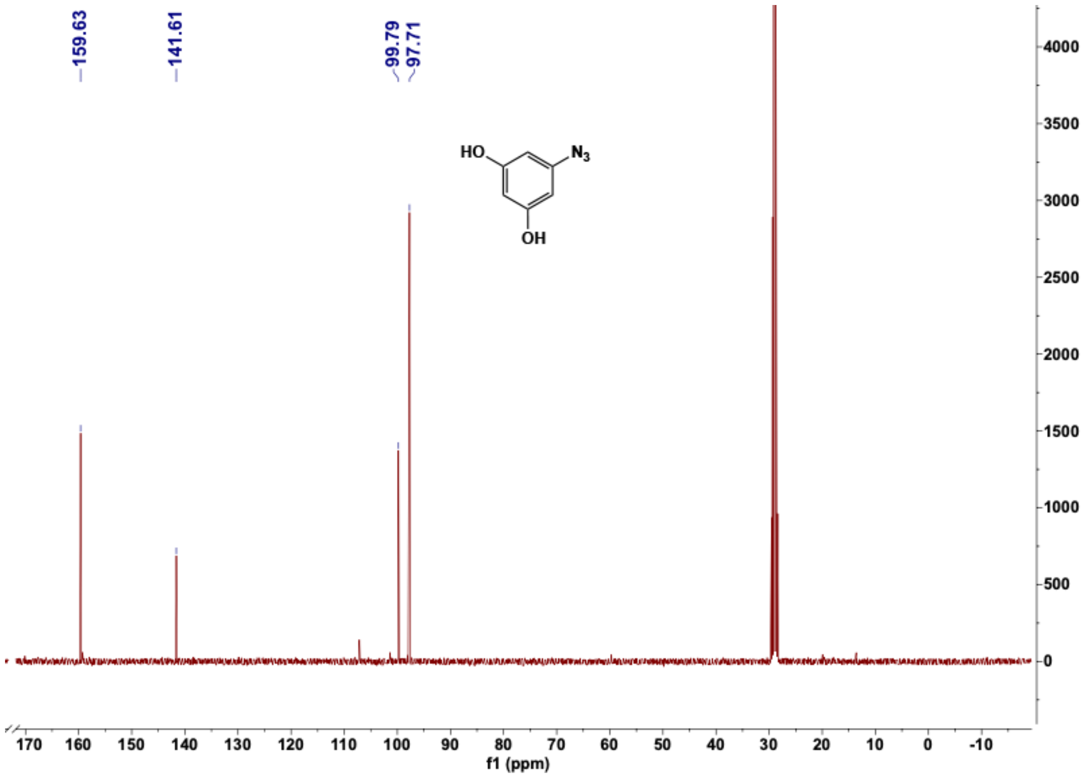

Supplementary Fig. 58. ^{13}C NMR spectrum of 5-azidobenzene-1, 3-diol, **Pd1** (*d*-acetone).

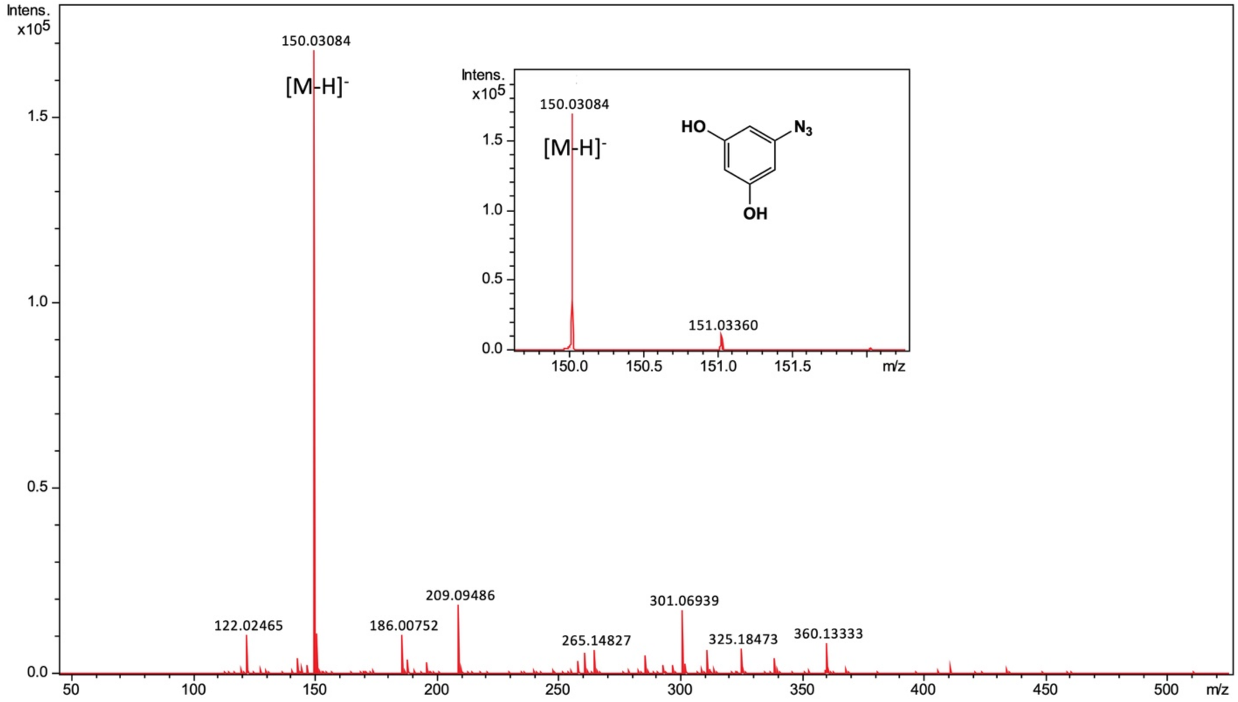

Supplementary Fig. 59. LC-MS spectrum of 5-azidobenzene-1, 3-diol, **Pd1**.

4-ethynylphenol (Pd2)

Ethynyltrimethylsilane (6.72 mmol) was added to a solution of 4-iodophenol (4.64 mmol), Pd $(PPh_3)_2Cl_2$ (0.14 mmol), and CuI (0.14 mmol) in 15 mL Et_3N, and the mixture was refluxed at 80 °C for 3 h under nitrogen. The solution was then cooled to room temperature, filtered, and concentrated in vacuum, and the crude product was chromatographed with Hexane/EtOAc as eluant, to obtain the precursor of compound (**Pd2**), trimethylsilyl ethynyl phenol (0.462 mmol, quantitative) as a brown oil. 0.2 mL Aqueous NaOH (5 M) was added to a solution of 4-((trimethylsilyl)ethynyl) phenol (0.274 mmol) in 10 mL MeOH, and the reaction solution was stirred for 3 h at room temperature under nitrogen. Then the solution was neutralized with conc. HCl and extracted with DCM (3 × 20 mL). The organic layers were combined, washed with brine (1 × 20 mL), dried over anhydrous Na_2SO_4, and concentrated under a vacuum. The crude product was chromatographed with Hexane/EtOAc as eluant, to afford the ethynylphenol as a dark red solid[16]. Yield: 50%.

^{1}H NMR (400 MHz, $CDCl_3$). δ 7.36-7.38 (d, J = 8.7 Hz, 2 H), 6.78-6.81(d, J = 8.7 Hz, 2 H), 5.4 (br, 1 H), 2.98 (s, 1 H).

^{13}C NMR (400 MHz, $CDCl_3$). δ 161.17, 131.20, 115.78, 77.35, 76.72.

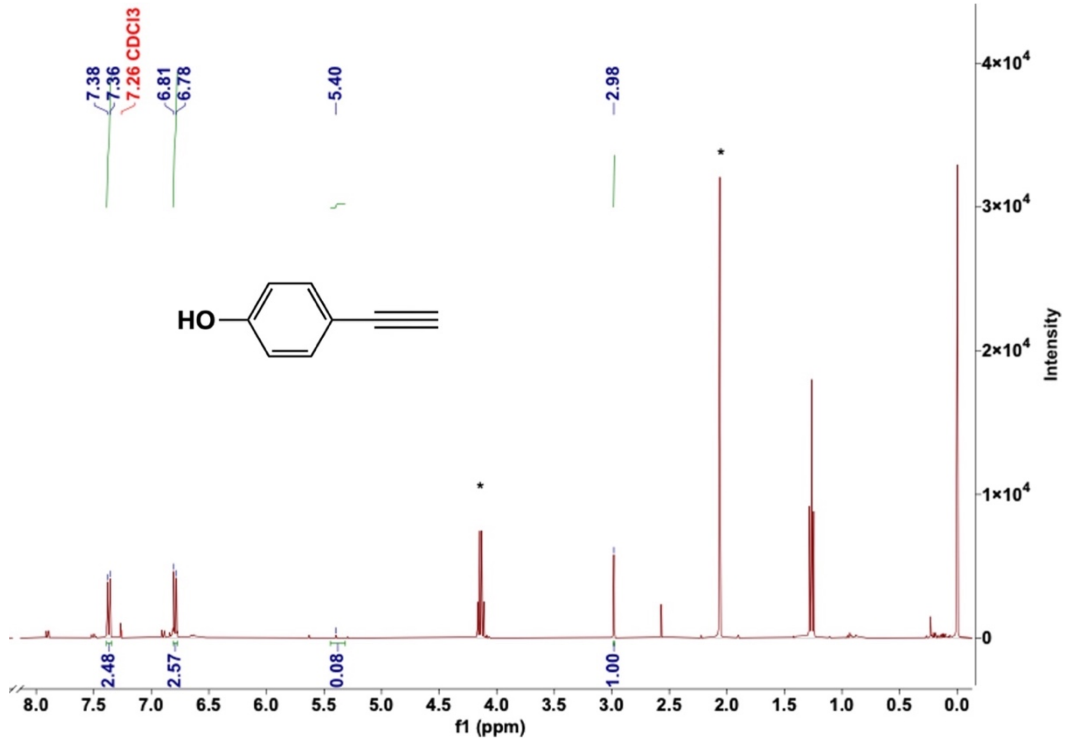

Supplementary Fig. 60. ^{1}H NMR spectrum of 4-ethynylphenol, **Pd2**. (*d*-$CDCl_3$).

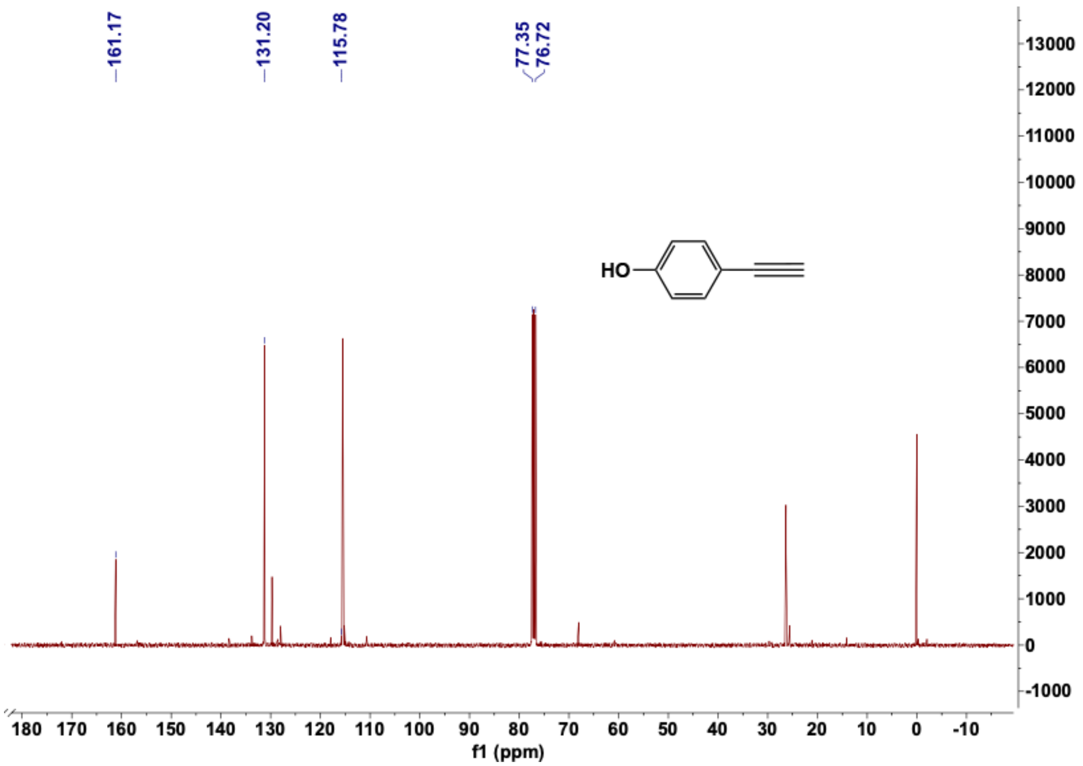

Supplementary Fig. 61. ^{13}C NMR spectrum of 4-ethynylphenol, **Pd2**. (d-$CDCl_3$).

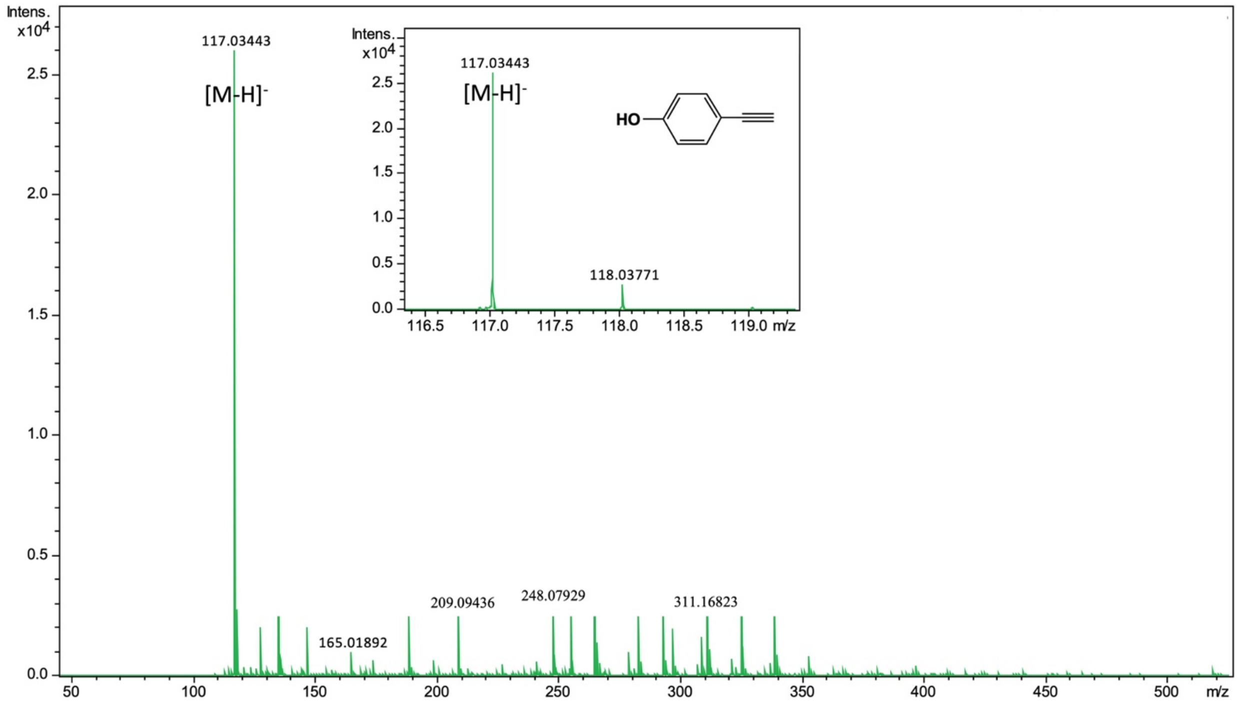

Supplementary Fig. 62. LC-MS spectrum of 4-ethynylphenol, **Pd2**.

5-(4-(4-hydroxyphenyl)-1H-1,2,3-triazol-1-yl) benzene-1,3-diol (Cd)

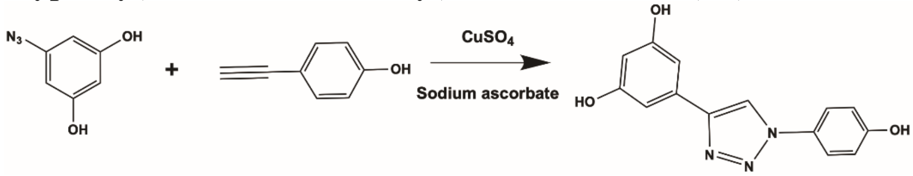

The synthesis was carried out with the following conditions: the overall volume in each test tube was 2 mL (H_2O: t-ButOH=1:1), containing a solution of azide, **Pd1**, (1 eq), alkyne, and **Pd2**, (1 eq). Freshly prepared sodium ascorbate solution (0.5 eq) was added, followed by $CuSO_4{\cdot}5H_2O$ (0.05 eq). The reaction was allowed to vigorously stir for 6 h at room temperature. After being diluted with water, the reaction mixture was cooled in ice. The precipitate was collected by filtration, washed with diethyl ether, and dried under a vacuum to afford a solid. Yield: 60%.

^{1}H NMR (400 MHz, DMSO-d_6) δ10.40 (s, 1 H), 9.65 (s, 4 H), 7.84 (s, 2 H),7.80 (d, J = 8.5 Hz, 2 H), 6.81-6.84 (d, J = 8.5 Hz, 2 H), 6.02 (s, 2 H).

^{13}C NMR (400 MHz, DMSO-d_6) δ 162.44, 159.94, 141.21, 133.74, 131.19, 129.04, 115.62, 100.22, 97.67.

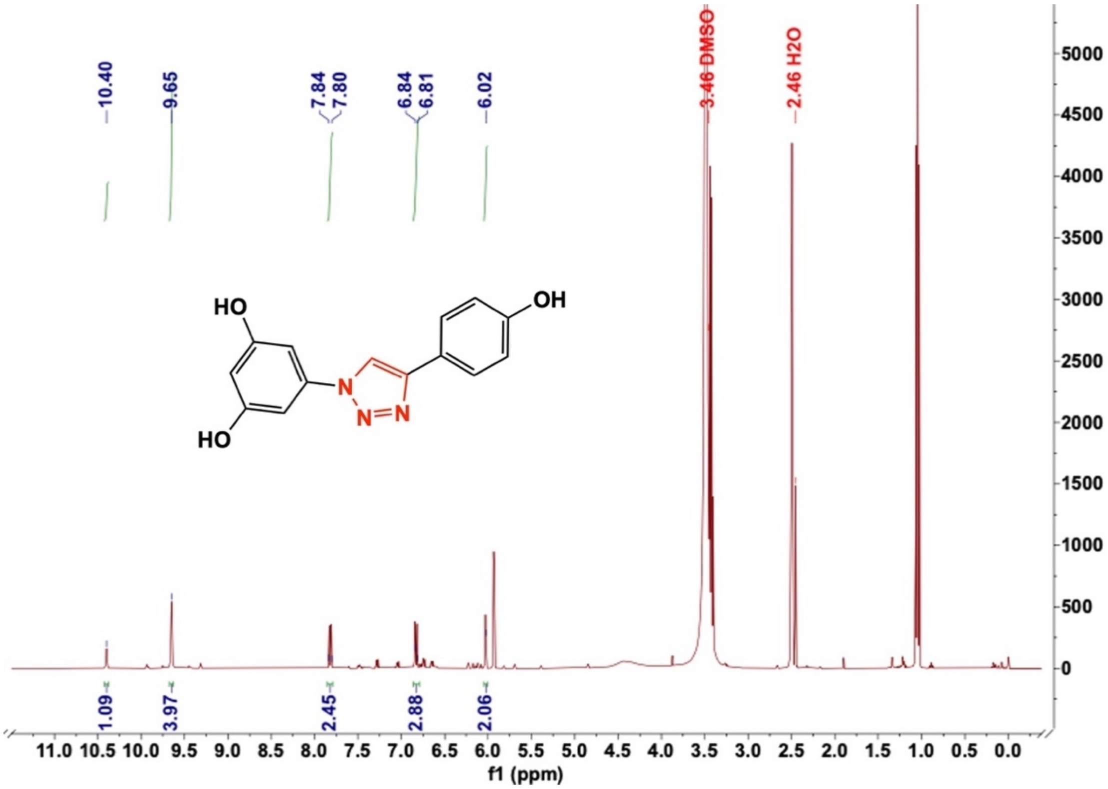

Supplementary Fig. 63. ^{1}H NMR spectrum of 5-(4-(4-hydroxyphenyl)-1H-1, 2, 3- triazol-1-yl) benzene-1, 3-diol, **Cd**. (DMSO-d_6, δ = 2.5).

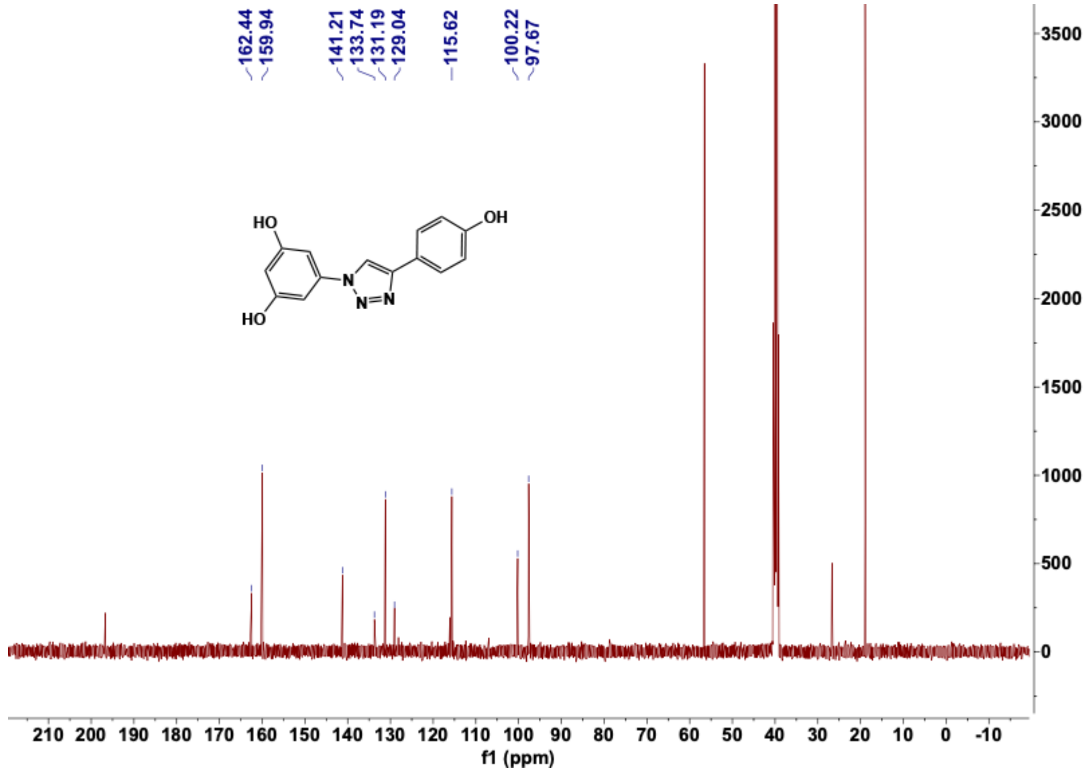

Supplementary Fig. 64. ^{13}C NMR spectrum of 5-(4-(4-hydroxyphenyl)-1H-1, 2, 3- triazol-1-yl) benzene-1, 3-diol, **Cd**. (DMSO-d_6, $\delta = 38.5$).

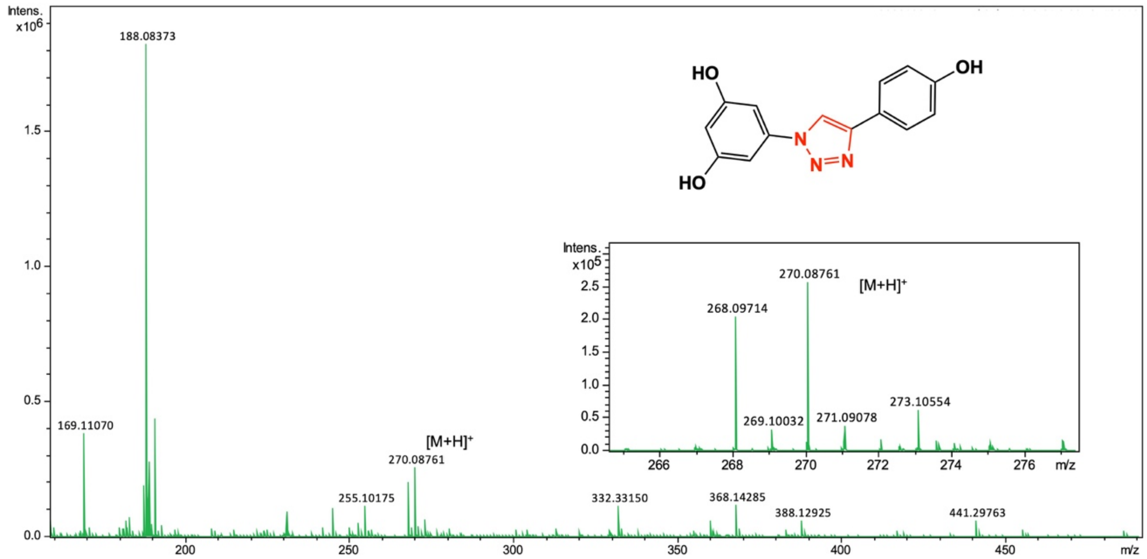

Supplementary Fig. 65. LC-MS spectrum of 5-(4-(4-hydroxyphenyl)-1H-1, 2, 3- triazol-1-yl) benzene-1, 3-diol, **Cd**.

References

1. Trott, O. & Olson, A.J. AutoDock Vina: Improving the speed and accuracy of docking with a new scoring function, efficient optimization, and multithreading. *J. Comput. Chem.* **31**, 455-461 (2010).
2. Schneidman-Duhovny, D., Inbar, Y., Nussinov, R. & Wolfson, H.J. PatchDock and SymmDock: servers for rigid and symmetric docking. *Nucleic Acids Res.* **33**, W363-W367 (2005).
3. Bai, N. et al. Rationalizing PROTAC-Mediated Ternary Complex Formation Using Rosetta. *J. Chem. Inf. Model.* **61**, 1368-1382 (2021).
4. Salomon-Ferrer, R., Case, D.A. & Walker, R.C. An overview of the Amber biomolecular simulation package. *Wiley Interdiscip. Rev.: Comput. Mol. Sci.* **3**, 198-210 (2013).
5. Maier, J.A. et al. ff14SB: Improving the Accuracy of Protein Side Chain and Backbone Parameters from ff99SB. *J. Chem. Theory Comput.* **11**, 3696-3713 (2015).
6. Caricato, M., Frisch, M.J., Hiscocks, J. & Frisch, M.J. Gaussian 09: IOps Reference. (Gaussian Wallingford, CT, USA, 2009).
7. Cornell, W.D., Cieplak, P., Bayly, C.I. & Kollman, P.A. Application of RESP charges to calculate conformational energies, hydrogen bond energies, and free energies of solvation. *J. Am. Chem. Soc.* **115**, 9620-9631 (1993).
8. Wang, J., Wang, W., Kollman, P.A. & Case, D.A. Automatic atom type and bond type perception in molecular mechanical calculations. *J. Mol. Graphics Modell.* **25**, 247-260 (2006).
9. Darden, T., York, D. & Pedersen, L. Particle mesh Ewald: An N·log(N) method for Ewald sums in large systems. *J. Chem. Phys.* **98**, 10089-10092 (1993).
10. Gowers, R.J. et al. Medium: ED; Size: 98 (United States; 2019).
11. Rodriguez, A. & Laio, A. Clustering by fast search and find of density peaks. *Science* **344**, 1492-1496 (2014).
12. Hélio Amante Miot, Gabrielli Brianezi2, Andréia de Almeida Tamega & Miot, L.D.B. Techniques of digital image analysis for histological quantification of melanin. *An Bras Dermatol.* **87**, 608-611 (2012).
13. Raina, K. et al. PROTAC-induced BET protein degradation as a therapy for castration-resistant prostate cancer. *Proc. Natl. Acad. Sci. U. S. A.* **113**, 7124-7129 (2016).
14. Zengerle, M., Chan, K.H. & Ciulli, A. Selective Small Molecule Induced Degradation of the BET Bromodomain Protein BRD4. *ACS Chem Biol* **10**, 1770-1777 (2015).
15. Andrus, M.B., Liu, J., Meredith, E.L. & Nartey, E. Synthesis of resveratrol using a direct decarbonylative Heck approach from resorcylic acid. *Tetrahedron Lett.* **44**, 4819-4822 (2003).
16. Hudson, S.A. et al. Application of Fragment Screening and Merging to the Discovery of Inhibitors of the Mycobacterium tuberculosis Cytochrome P450 CYP121. *Angew. Chem., Int. Ed.* **51**, 9311-9316 (2012).
