# Supplementary material for: Endogenous tyrosinase-catalyzed therapeutics
Source: Nat Commun. 2025 Jul 12;16:6463. doi: 10.1038/s41467-025-61799-7 (PMC12255739; doi:10.1038/s41467-025-61799-7)
Supplement: Supplementary file 2 — Reporting Summary [file 41467_2025_61799_MOESM2_ESM.pdf]

nature portfolio

Corresponding author(s): Quanyin Hu

Last updated by author(s): Jun 16, 2025

Reporting Summary

Nature Portfolio wishes to improve the reproducibility of the work that we publish. This form provides structure for consistency and transparency in reporting. For further information on Nature Portfolio policies, see our Editorial Policies and the Editorial Policy Checklist.

Statistics

For all statistical analyses, confirm that the following items are present in the figure legend, table legend, main text, or Methods section.

n/a	Confirmed	
☐	☒	The exact sample size (n) for each experimental group/condition, given as a discrete number and unit of measurement
☐	☒	A statement on whether measurements were taken from distinct samples or whether the same sample was measured repeatedly
☐	☒	The statistical test(s) used AND whether they are one- or two-sided<br>*Only common tests should be described solely by name; describe more complex techniques in the Methods section.*
☐	☒	A description of all covariates tested
☐	☒	A description of any assumptions or corrections, such as tests of normality and adjustment for multiple comparisons
☐	☒	A full description of the statistical parameters including central tendency (e.g. means) or other basic estimates (e.g. regression coefficient) AND variation (e.g. standard deviation) or associated estimates of uncertainty (e.g. confidence intervals)
☐	☒	For null hypothesis testing, the test statistic (e.g. F, t, r) with confidence intervals, effect sizes, degrees of freedom and P value noted<br>*Give P values as exact values whenever suitable.*
☒	☐	For Bayesian analysis, information on the choice of priors and Markov chain Monte Carlo settings
☒	☐	For hierarchical and complex designs, identification of the appropriate level for tests and full reporting of outcomes
☒	☐	Estimates of effect sizes (e.g. Cohen's d, Pearson's r), indicating how they were calculated

Our web collection on statistics for biologists contains articles on many of the points above.

Software and code

Policy information about availability of computer code

Data collection	1H NMR spectrum and 13C NMR spectrum were recorded via a Bruker Avance III HD 400 MHz instrument (AV-400) NMR instrument. Liquid Chromatography Mass Spectra (LCMS) measurements were performed on a Bruker MaXis time-of-flight spectrometer. Fluorescence spectra were detected by a QuantaMaster Model C-60/2000 Spectrofluorimeter. A Nikon Intensilight Fluorescence Microscope acquired the bright field microscopy characterization. Agilent Infinity II measured the high-performance liquid chromatography (HPLC) analysis. A Nikon AXR Confocal Microscope acquired the confocal laser scanning microscopy (CLSM) characterization. The flow cytometry data were detected by the ThermoFisher Attune Cell Analyzer.
Data analysis	Prism 10 were used for statistical analysis. FlowJo_V10. was used for analysis of Flow Cytometer. PyMOL was used for docking analysis. Iamge J was used for analysis west bolt.

For manuscripts utilizing custom algorithms or software that are central to the research but not yet described in published literature, software must be made available to editors and reviewers. We strongly encourage code deposition in a community repository (e.g. GitHub). See the Nature Portfolio guidelines for submitting code & software for further information.

Data

Policy information about availability of data

All manuscripts must include a data availability statement. This statement should provide the following information, where applicable:

- Accession codes, unique identifiers, or web links for publicly available datasets
- A description of any restrictions on data availability
- For clinical datasets or third party data, please ensure that the statement adheres to our policy

The authors provide all supporting data for this study in the article and the Supplementary Information. Source data are provided with this paper.

Research involving human participants, their data, or biological material

Policy information about studies with human participants or human data. See also policy information about sex, gender (identity/presentation), and sexual orientation and race, ethnicity and racism.

Reporting on sex and gender	N/A
Reporting on race, ethnicity, or other socially relevant groupings	N/A
Population characteristics	N/A
Recruitment	N/A
Ethics oversight	N/A

Note that full information on the approval of the study protocol must also be provided in the manuscript.

Field-specific reporting

Please select the one below that is the best fit for your research. If you are not sure, read the appropriate sections before making your selection.

[x] Life sciences [ ] Behavioural & social sciences [ ] Ecological, evolutionary & environmental sciences

For a reference copy of the document with all sections, see nature.com/documents/nr-reporting-summary-flat.pdf

Life sciences study design

All studies must disclose on these points even when the disclosure is negative.

Sample size	All data were obtained from a minimum of three independent experiments and were presented as the mean ± standard deviation (SD) or mean ± standard error (SE). Statistical evaluation was analyzed using GraphPad Prism. Two-group comparisons were analyzed with the unpaired Student's t-test. Analysis of variance (ANOVA) was used to compare multiple groups (> two groups) followed by Dunnett's multiple comparisons test. (*P < 0.05, **P < 0.01, ***P < 0.001, ****P < 0.0001).
Data exclusions	No data were excluded from the analysis.
Replication	Experiments are repeated at least three time unless otherwise unless otherwise stated in the respective figure legend. Experimental findings are reproducible.
Randomization	The samples were divided into different groups randomly in all experiments.
Blinding	The investigator are not blinded for most of experiments since the experimental design, execution, and data analysis are performed by the same person. Tumor size measurement are conducted by an independent operator, who is unaware of the treatment conditions.

Reporting for specific materials, systems and methods

We require information from authors about some types of materials, experimental systems and methods used in many studies. Here, indicate whether each material, system or method listed is relevant to your study. If you are not sure if a list item applies to your research, read the appropriate section before selecting a response.

Materials & experimental systems

n/a	Involved in the study	
☐	☒	Antibodies
☐	☒	Eukaryotic cell lines
☒	☐	Palaeontology and archaeology
☐	☒	Animals and other organisms
☒	☐	Clinical data
☒	☐	Dual use research of concern
☒	☐	Plants

Methods

n/a	Involved in the study	
☒	☐	ChIP-seq
☐	☒	Flow cytometry
☒	☐	MRI-based neuroimaging

Antibodies

Antibodies used	The mouse monoclonal IgG1 κ anti-VHL antibody (sc-135657,1:1000).<br>Tyrosinase Recombinant Rabbit Monoclonal Antibody (JA52-11,1:1000).<br>MITF (D5G7V) Rabbit mAb #12590.(1:1000)<br>Anti-β-actin (ab4990,1:500000).<br>goat anti-rabbit IgG H&L (HRP) secondary antibodies (1:10,000)
Validation	All antibodies used in this manuscript are commercially available. The validation and quality control are performed by the corresponding vendors.

Eukaryotic cell lines

Policy information about cell lines and Sex and Gender in Research

Cell line source(s)	The murine cell line B16F10 and human cell line A375 are purchased from ATCC. The primary human melanocytes are gifted by the laboratory of Dr. Vijayasaradhi Setaluri from the Department of Dermatology, the University of Wisconsin-Madison.
Authentication	The cell lines were morphologically confirmed according to the information provided by ATCC.
Mycoplasma contamination	Cell lines are routinely tested for potential mycoplasma contamination.
Commonly misidentified lines (See ICLAC register)	None of these cell lines were used.

Animals and other research organisms

Policy information about studies involving animals; ARRIVE guidelines recommended for reporting animal research, and Sex and Gender in Research

Laboratory animals	The male C57BL/6 mice (6-8 weeks) and male nude mice (5-6 weeks) are purchased from the Jackson Laboratory. All mice were housed in a specific pathogen-free environment at 20±3 ℃ and 50±5% humidity, with a 12h light-dark cycle.
Wild animals	The study did not involve wild animals.
Reporting on sex	Sex of mice was not considered in this study.
Field-collected samples	The study did not involve samples collected from the field.
Ethics oversight	The animal study protocol was approved by the Institutional Animal Care and Use Committee (IACUC) at the University of Wisconsin-Madison.

Note that full information on the approval of the study protocol must also be provided in the manuscript.

Plants

Seed stocks	N/A
Novel plant genotypes	N/A
Authentication	N/A

Flow Cytometry

Plots

Confirm that:

- [x] The axis labels state the marker and fluorochrome used (e.g. CD4-FITC).
- [x] The axis scales are clearly visible. Include numbers along axes only for bottom left plot of group (a 'group' is an analysis of identical markers).
- [x] All plots are contour plots with outliers or pseudocolor plots.
- [x] A numerical value for number of cells or percentage (with statistics) is provided.

Methodology

Sample preparation	B16F10 and A375 cells were plated in 6-well plates.Next, azide coumarin (pS1,10 µM) and alkyne (pS2,10 µM) were added and incubated with the cells for 12 h. After being washed with PBS for 2 times, the cells were harvested by trypsin and resuspended in PBS. The intracellular fluorescence of product was analyzed using flow cytometry (450/50). For analysis of prodrug activation-induced apoptosis,DR-A375 and DR-B16F10 cells, seeded at 2 × 10^5 cells/well in 6-well plates, were incubated for 12 h before treatment with 100 µM Pd1, Pd2, Cd, Pd1 + Pd2, and pS1 + pS2 + SA for 24 h. Following the treatment phase, cells underwent staining with Annexin V (5 µl) in 100 µl binding buffer for 15 minutes at room temperature in darkness. PI (1 µl) in 100 µl binding buffer was added for 15 minutes without rinsing. Flow cytometry was employed to assess Annexin V-PI staining, using fluorescein isothiocyanate–Annexin V (Ex = 488 nm, Em = 520/21 nm) and PI (Ex = 561, Em = 590/30 nm).
Instrument	ThermoFisher Attune Flow Cytometer.
Software	FlowJo software package.
Cell population abundance	The absolute cells around 10000 were analyzed for each group.
Gating strategy	Initial cell populations were gated for a live population using FSC and SSC plot of cell only sample. The gate was set to remove cell debris and dead cells.

- [x] Tick this box to confirm that a figure exemplifying the gating strategy is provided in the Supplementary Information.
